# Historical trends of heavy metals applying radio-dating and neutron activation analysis (NAA) in sediment cores, Burullus Lagoon, Egypt

**DOI:** 10.1007/s11356-024-33761-5

**Published:** 2024-06-13

**Authors:** Alia Ghanem, Afaf Nada, Hosnia Abu-Zeid, Waiel Madcour, Said A. Shetaia, Noha Imam

**Affiliations:** 1 Physics Department, Faculty of Women for Arts, Science & Education, Ain Shams University, Cairo, Egypt; 2 Radiation Protection Department, Nuclear Research Center, Egyptian Atomic Energy Authority, Cairo, Egypt; 3 Geology Department, Faculty of Science, Al-Azhar University, Cairo, Egypt; 4 Physics & Geology Lab, National Institute of Oceanography and Fisheries, Cairo, Egypt

**Keywords:** Fallout radionuclides, Gamma spectrometry, Radiometric dating, Sedimentation rates, Sediment chronology, Pollution indices

## Abstract

Burullus lagoon is part of Egypt’s protected area network. The lagoon serves as a reservoir for drainage water discharged from agricultural areas, and the lake’s sediments provide a unique opportunity to record environmental behavior and reconstruct of the heavy metal contamination history. In the present study, the sediment chronology, sedimentation rates, and metal accumulation fluxes were estimated in four sediment cores using ^210^Pb dating models to evaluate how human activities have affected the coastal environment. Using the radioisotopes ^210^Pb and ^137^Cs, radiometric dating was carried out using gamma-ray spectrometry. At the Egypt Second Research Reactor (ETRR-2), the element concentrations were determined using the instrumented neutron activation analysis (INAA- k_0_ method). Our findings show that the constant rate of supply (CRS), which has been verified with the peak of artificial radionuclide ^137^Cs, is the best model performed for the chronology of Burullus Lagoon. The average sedimentation rate, according to ^210^Pb dating models, is 0.85 cm/year. The large variation in sedimentation rates, especially after the 1990s, is consistent with an increase in the anthropogenic flux of heavy metals. This may be led into a significant environmental problem such as reducing the size of the lake and degrading the quality the water in Burullus Lagoon. Enrichment factor (EF) of the studied elements displayed the following order: Cl > Ca > Na > Br > Zn > Ta > Ti > V > Cr > Sc > Mg > Mn > Fe > Hf which is higher than unity. Furthermore, the Nemerow pollution index (PI _Nemerow_) revealed that pollution was increasing in the direction of the drains and slightly polluted. Consequently, pollutant indices showed that urbanization and industrial development may have increased the depositional fluxes of the metals in sediments over time.

**Supplementary Information:**

The online version contains supplementary material available at 10.1007/s11356-024-33761-5.

## Introduction

Lake sediment cores supply a useful information on anthropogenic and natural inputs, the history of sedimentation, and the sources of pollution (Lin et al. 2018; Pappa et al. 2018). Natural environmental archives can be used to manage processes, identify pertinent sources, reconstruct temporal trends in concentrations, and evaluate the pollution state of ecosystems. Sediment chronologies are essential for the interpretation of natural ecosystems, such as lake sediments, which have been used to track atmospheric contamination such as heavy metals, organic contaminants, and radioactive releases from nuclear power plants as well as to assess the past history of changes in lake water quality related to phenomena like acid rain (Appleby 2001; Palani et al. 2023). Furthermore, it plays a significant role in the investigation of physico-chemical processes (e.g., bioturbation, early diagenesis), the study of geological phenomena (e.g., floods, landslides), and the determination of marine radiomodeling variables (e.g., vertical dispersion velocity, radionuclides’ diffusion coefficient in the sediment) (Eleftheriou et al. 2018; Pappa et al. 2018, 2019). The radiochronology of sediments is largely based on the vertical profiles of radionuclides in this frame. Combining two types of fallout radionuclides man-made ^137^Cs ${(T}_{1/2}=30.07$ a) and natural ^210^Pb (${T}_{1/2}= 22.3$ a) has shown to be a valuable method for calculating sediment/mass accumulation rates and understanding sediment geochronology in different lakes (Appleby 2001; Semertzidou et al. 2019; Imam and Salem 2023). Sediment dating with ^210^Pb, which has been successfully used to reconstruct a variety of environmental processes linked to climatic change, is the most popular method for determining recent chronologies (100–150 year) and aquatic ecosystem sedimentation rates (Barsanti et al. 2020). Three models of ^210^Pb have been developed to balance the diverse influences of human activity, geology, and sedimentary processes (Appleby and Oldfield 1992). The three models are known by the acronyms constant rate of supply (CRS), constant flux constant sedimentation (CFCS), and constant initial concentration (CIC). Based on various environmental factors, each model has a unique set of assumptions.

The CIC model assumes a uniform and monotonic decrease in ^210^Pb_ex_ activity with depth, which means that the CIC model predicts younger dates for more active deeper layers, which is physically impossible (Zhang et al. 2015a). In the CFCS model, ^210^Pb_ex_ activity decreases exponentially with dry mass accumulation when the ^210^Pb_ex_ flux and sedimentation rate remain constant (Appleby and Oldfield 1983). Because the CIC and CFCS models are strongly influenced by the initial ^210^Pb concentration, these models cannot provide reliable chronology. Conversely, the CRS model is not sensitive to these changes in the initial concentration and sedimentation rate of different layers. The sedimentation processes in each aquatic ecosystem should be taken into consideration when choosing models, as each one makes specific assumptions based on various environmental conditions (Guo et al. 2020). The different dating models were used for the same cores with the ^137^Cs event marker to confirm the dating estimates of the sediment records and to circumvent the discrepancy between the three models (Appleby 2008; Putyrskaya et al. 2015). According to previous (Eleftheriou et al. 2018; Pappa et al. 2018, 2019), the above models can be used to reconstruct historical pollution events associated with environmental phenomena such as strong earthquakes, volcanic eruptions, and hydrological changes, as well as industrial. The man-made radioisotope ^137^Cs, a well-known chronostratigraphic marker, was created in nuclear fission events and largely discharged into the environment during nuclear weapon testing in the 1950s and 1960s, particularly the 1963 fallout maximum (Kumar et al. 2014; Brandon Michael Boyd 2016). Indeed, the majority of studies on ^137^Cs distribution as an artificial radionuclide in the Mediterranean Sea have been attributed to global fallout from nuclear weapons tests, the Chernobyl accident, and the nuclear industry (Iridoy 2017), and furthermore, the nuclear accident at the Fukushima Daiichi (FD) nuclear disaster in Japan (2011) (Stäger et al. 2023). The FD accident, caused by the tsunami resulting from the Tohoku earthquake on 11 March 2011, involves a large release of radioisotopes into the environment (Bin Feng et al. 2022; Stäger et al. 2023). In recent sedimentary deposits, the use of ^137^Cs dating as a complementary technique to ^210^Pb dating has become increasingly important for determining age-depth models (Sarı et al. 2018; Shah et al. 2020).

Lake pollution is one of the biggest environmental problems caused by various sorts of pollutants in many parts of the world, and it poses a significant threat to the planet’s freshwater supplies (Vörösmarty et al. 2010). Sediment pollution in lakes is a result of both anthropogenic and natural processes (Li et al. 2013). These include natural processes such as soil and rock degradation, erosion, wildfires and volcanic eruptions, and anthropogenic processes such as industrial discharges, mining and refining processes, agricultural runoff, wildlife runoff, domestic wastewater, and atmospheric deposition from burning fossil fuels (Davutluoglu et al. 2011). Indeed, anthropogenic sources strongly influence heavy metal accumulation in the marine environment (Perumal et al. 2021). Due to their toxicity and insolubility, heavy metals are one of the emerging environmental contaminants that should be concerned us in aquatic environments (Kumar et al. 2019). Heavy metals in aquatic systems have multiple sources. Bedrock weathering, erosion, and leaching are natural sources; anthropogenic sources include sewage, industrial discharges, fertilizers, and agricultural activities (Kostka and Leśniak 2020, 2021). Therefore, it is important to understand how pollutants are deposited, transformed, and remobilized over time and under changing environmental conditions (such as climate change). Several techniques can be used for element analysis, including instrumented nuclear activation analysis (INNA), X-ray fluorescence (XRF), atomic absorption spectroscopy (AAS), and inductively coupled plasma mass spectrometry (ICP-MS). Activation analysis is an elemental determination method based on nuclear reactions in which stable nuclei are converted into other, usually radioactive nuclei, and the reaction products are then measured (Greenberg et al. 2011). INAA is the most commonly used method due to its small sample size, high sensitivity, selectivity, non-destructive analysis, sample matrix independence, and ability to simultaneously identify multiple elements in the same sample. A variety of indices such as the enrichment factor (EF), ecological risk factor (Er), and Nemerow pollution index (PI _Nemerow_) have been used to measure the ecotoxicological hazard of contaminants in the sediment cores. The enrichment factor was determined for all examined elements to discriminate between anthropogenic and natural elemental sources.

The Nile Delta’s Manzala, Burullus, Edku, and Mariut lagoons are a major source of water for thousands of Egyptians (Younis 2019). These lakes began to receive a sizable amount of drainage water from agricultural activities after the Aswan High Dam was built in 1964, in addition to seasonal overflow of saltwater. In recent decades, urbanization and industrialization have contributed to an increase in pollutants from a variety of sources, such as industry, agriculture, and waste water (Negm et al. 2019). Burullus Lagoon is among the coastal lagoons that are most at risk because of the abundance of aquatic vegetation, overfishing, an increase in fish farming, and agricultural drainage (Khalil and El-Gharabawy 2016). The main factors contributing to the rise in the concentrations of heavy metals in lake water are increased irrigation, effluent from near-shore aquaculture ponds, and untreated domestic sewage discharge (Al-Afify et al. 2023). In order to better assess and manage the entire ecosystem, it is possible to identify the processes that contribute to the degradation of wetlands as well as the sources of pollution through studies of the spatial and temporal distribution of heavy metals in the delta banks. A thorough analysis is urgently required to determine whether these levels are the result of exposure to contaminants in sediment that have accumulated in the past or inflow from nearby anthropogenic sources of pollution.

Therefore, the purpose of the investigation was to analyze sediment core samples from the Burullus lagoon to achieve the following goals: (i) determine the sediment chronology and sedimentation rates using different models of ^210^Pb; (ii) reconstruct historical trends and fluxes of anthropogenic metal into the lake and the influence of the catchment area; and (iii) determine pollution indices to evaluate the levels of contamination with heavy metals. We hope that the results of this research will aid in making decisions on the future structure and distribution of Burullus Lagoon.

## Material and procedures

### Research area

The second largest of the northern lagoons on Egypt’s Mediterranean coast, Burullus Lagoon, is a UNESCO-protected area. It has a surface area of about 410 km^2^ and is one of four lagoons with shallow brackish water. It is situated along the Rosetta Nile bend, in the middle of Egypt’s Mediterranean coast, between 31° 22′ and 31° 26′ North and 30° 30′ and 31° 10′ East longitudes. Boughaz El-Burullus, which is in the northeastern corner of the lagoon and has depths ranging from 0.4 to 2.00 m, is the passage that connects the lagoon to the Mediterranean. Thirty-four percent of the lagoon’s surface is made up of islands and floating vegetation. In the western region of Burullus Lagoon, there is the Brimbal Canal, which is a source of fresh water, and numerous agricultural drains that empty into the lake (Negm et al. 2019). The Burullus Lagoon has recently received drainage water from a number of drains, including drains 7, 8, 9, 11, Nasser, El-Gharbiya, and El-Burullus, totaling about 3900 Mm^3^/year (Shalby et al. 2020; Shetaia et al. 2022). There are many serious problems affecting the lakes in Egypt’s northern Nile Delta, including an increase in the flow of fresh water, pollution, lake degradation, rapid population growth, and erosion rates (Younis 2019).

### Field sampling

**Fig. 1:**
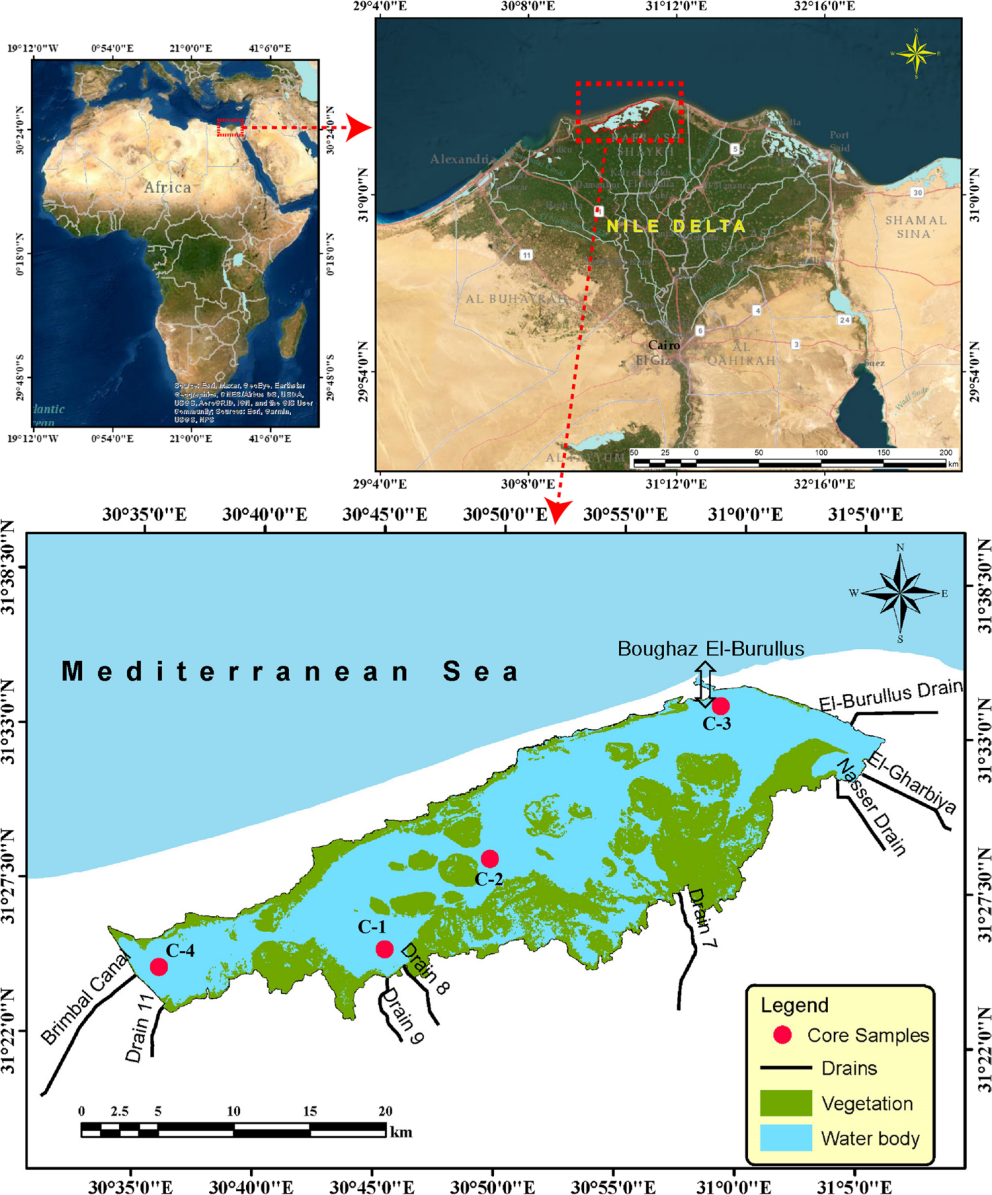
Map of Burullus Lagoon and sampling sites

In 2018, four sediment cores from the Burullus lagoon were collected, as shown in Table 1 and Fig. 1. The maps were produced using the ArcGIS program (version 10.8) (ESRI, CA, USA). Core sediments were taken by manually taking a sample inside a plastic tube that was 2 to 3 m long and 10 cm in diameter. The sediment samples were kept in a refrigerator at 4 °C until they were opened for analysis. Glass and plastic were chosen as the material types to avoid contaminating the samples with metal. Before subsamples were collected for additional analysis, the central tubes were cut lengthwise, opened with nylon string, described, and photographed in the lab. The collected sediment cores were cut into 4-cm-long slices for the analysis of radioactivity and heavy metals.

**Table 1:** Location of sampling sites of Burullus lagoon

Core profile	Sample date collection	Coordination		Length cm
		Longitude	Latitude	
C-1	2018	30.7656002	31.4162998	56
C-2		30.8225002	31.4762993	56
C-3		30.9789009	31.5697002	48
C-4		30.5893002	31.4039001	48

### Radiometric dating models based on ^210^Pb

The sediment cores were analyzed using the ^210^Pb models to determine the mass accumulation rate (MAR, (r), g/cm year) and sediment accumulation rate (SAR, (s), cm/year). The artificial radionuclide ^137^Cs (T_1/2_ = 30.08 a) was used as an independent constraint on the sediment ages for the 2011 and 1986 events in order to validate these models. The ^210^Pb_ex_ was used as the main parameter in the ensuing models. It was calculated by subtracting the actual ^226^Ra activity from the total ^210^Pb activity.

#### Constant flux constant sedimentation

In the CFCS model, the mass accumulation rate and ^210^Pb flux are treated as constants (Appleby and Oldfield 1983). The slope of the regression line can be utilized to determine the mass accumulation rate because the ^210^Pb_ex_ logarithm against cumulative mass depth will most closely reflect a straight line (Guo et al. 2020).

1 $$r={~}^{-\lambda }\!\left/ \!{~}_{slope}\right.$$

Calculating the uncertainty (U) in the mass accumulation rate can be done using the equation below (Sanchez-Cabeza and Ruiz-Fernández 2012):

2 $$U\left(r\right)=r\times {\left({\left[{~}^{U(slope)}\!\left/ \!{~}_{slope}\right.\right]}^{2}+{\left[{~}^{U(\lambda )}\!\left/ \!{~}_{\lambda }\right.\right]}^{2}\right)}^\frac{1}{2}$$

Additionally, by replacing the section depth (z_i_) for the cumulative mass depth (m_i_) in a linear regression analysis, it is feasible to determine the sediment accumulation rate (Bruel and Sabatier 2020).

3 $$\text{s}={~}^{-\uplambda }\!\left/ \!{~}_{\text{slope}}\right.$$

To determine the uncertainty (U) in the mass accumulation rate, apply the equation below:

4 $$U\left(s\right)=s\times {\left({\left[{~}^{U(slope)}\!\left/ \!{~}_{slope}\right.\right]}^{2}+{\left[{~}^{U(\lambda )}\!\left/ \!{~}_{\lambda }\right.\right]}^{2}\right)}^\frac{1}{2}$$

The CFCS model can be used to calculate the age (t) of the sediment at a depth (i) as follows:

5 $$t={~}^{{m}_{i}}\!\left/ \!{~}_{r}\right.$$

The uncertainty (U) in the sediment age may be calculated using the equation below.

6 $$U\left(t\right)=t\times {\left({\left[{~}^{U(r)}\!\left/ \!{~}_{r}\right.\right]}^{2}+{\left[{~}^{U({m}_{i})}\!\left/ \!{~}_{{m}_{i}}\right.\right]}^{2}\right)}^\frac{1}{2}$$

#### Constant rate of supply

The fundamental hypothesis of the CRS model is the ^210^Pb_ex_ flux to the sediment surface (Appleby et al. 2001). This depends on comparing the ^210^P_ex_ inventory in sediment cores at various depths to the overall ^210^P_ex_ inventory. According to the CRS model, the following equations can be used to compute the layer age (t_i_) at depth I, the MAR (r, g/cm year), the SAR (s, cm/year), and their uncertainty (U) (Sanchez-Cabeza and Ruiz-Fernández 2012; Loan et al. 2023).

7 $$t(i)=\left({~}^{1}\!\left/ \!{~}_{\lambda }\right.\right)\mathit{ln}\left[{~}^{A(0)}\!\left/ \!{~}_{A(i)}\right.\right]$$

8 $$U\left[(t(i)\right]=\frac{1}{\uplambda }\times {\left({\left(\text{U}\left(\uplambda \right) {\text{t}}_{\text{i}}\right)}^{2}+{\left(\frac{\text{U}\left(\text{A}\left(0\right)\right)}{A\left(0\right)}\right)}^{2}+\left[ \left(1-\frac{2A\left(i\right)}{A\left(0\right)}\right) {\left(\frac{\text{U}\left(\text{A}\left(\text{i}\right)\right)}{A\left(i\right)}\right)}^{2}\right]\right)}^{{~}^{1}\!\left/ \!{~}_{2}\right.}$$

9 $$r\left(i\right)={~}^{\lambda A\left(i\right)}\!\left/ \!{~}_{{C}_{i}}\right.$$

10 $$U\left(r(i)\right)=r(i)\times {\left({\left[{~}^{U(A\left(i\right))}\!\left/ \!{~}_{A(i)}\right.\right]}^{2}+{\left[{~}^{U(C\left(i\right))}\!\left/ \!{~}_{C(i)}\right.\right]}^{2}+{\left[{~}^{U(\lambda )}\!\left/ \!{~}_{\lambda }\right.\right]}^{2}\right)}^\frac{1}{2}$$

11 $$s\left(i\right) =\left[{~}^{r(i)}\!\left/ \!{~}_{\rho (i)}\right.\right]\times 100$$

12 $$U\left(s(i)\right)=s(i)\times {\left({\left[{~}^{U(r\left(i\right))}\!\left/ \!{~}_{r(i)}\right.\right]}^{2}+{\left[{~}^{U(\rho \left(i\right))}\!\left/ \!{~}_{\rho (i)}\right.\right]}^{2}\right)}^\frac{1}{2}$$

where $\rho \left(i\right)$ is a dry bulk density.

According to Binford (1990), the deeper core regions of the CRS model consistently display a too-old age mistake. The underestimating of ^210^Pb_ex_, which can be the result of analytical constraints, sampling design, or both, is what leads to the too-old age error (Bruel and Sabatier 2020). Tylmann et al. (2016) recommend that ^210^Pb_ex_ dating based on the CRS model be corrected by reference age to prevent too-old age mistakes for deeper core sections. If x_1_ is the depth of one reference point (the ^137^Cs peak marker) with a known age t_1_ in the core, the mean ^210^Pb flux (f) above the reference point can be calculated using the following equation (Appleby 2000, 2001; Chen et al. 2019).

13 $$F=\frac{\lambda \Delta A}{{e}^{-\lambda {t}_{1}}-{e}^{-\lambda {t}_{2}}}$$

where *ΔA* represents the total inventory of ^210^Pb_ex_ between reference depths x_1_ and x_2_, corresponding to age t_1_ and t_2_, respectively. The following equation can be used to determine sediment age (t) between ${\text{x}}_{1}$ and ${\text{x}}_{2}$ at any depth using the estimated average flux f(Zhang et al. 2015b):

14 $$t=\frac{-1}{\lambda } \mathit{ln}\left[\mathit{exp}\left(-\lambda {t}_{1}\right)-\Delta {A}_{{x}_{1}-x}\frac{\lambda }{f}\right]$$

The age was determined for the layers below the depth of the time marker using the following formula (Chen et al. 2019):

15 $$t={T}_{1}-\frac{1}{\lambda } \mathit{ln}\frac{{A}_{{x}_{1}}}{{A}_{x}}$$

where *T*_*1*_ is the chronostratigraphic date of the x_1_ depth and A_x1_ is the ^210^Pb_ex_ inventory below the x_1_ depth.

### Ecological assessment of heavy metals

A group of metrics known as pollution indices are used to evaluate the state of the environment and forecast how long pollution will last. The enrichment factor (EF), the Nemerow contamination index (PI Nemerow), and the environmental risk factor (Er) are a few examples of contamination indices.

#### Enrichment factor

One of the most popular ways to differentiate between anthropogenic and natural sources is the enrichment factor (Badawy et al. 2021). Commonly used reference elements for EF calculations are Ti, Zr, Fe, Al, and Sc (Ye et al. 2020). We chose Al as a normalizing element in this investigation for the following reasons: because (a) it is always found as a background metal in combination with other elements due to its uniform natural concentration and abundance in the Earth’s crust (Man et al. 2022); (b) it is largely generated from alumina-silicates (Wang et al. 2019). The following formula was used to compute the EF of heavy metals (Li et al. 2017):

16 $$EF= {~}^{{\left[{C}_{Metal}/{C}_{Al}\right]}_{sample}}\!\left/ \!{~}_{{\left[{C}_{Metal}/{C}_{Al}\right]}_{Background}}\right.$$

where ${\left[{C}_{Metal}/{C}_{Al}\right]}_{Background}$ and ${\left[{C}_{Metal}/{C}_{Al}\right]}_{sample}$ are the corresponding ratios of the concentrations of the element and the normalizing element (Al) in the background (in the continental shale abundance reported by (Li and Schoonmaker 2014)) and sample, respectively. The existence of pollution is indicated by an EF value greater than 1 (Li et al. 2022), and the following formula can be used to determine the anthropogenic contribution to the metals.

17 $${M}_{anthropogenic}={M}_{sample}{-Al}_{sample}\times {\left[M/Al\right]}_{Background}$$

#### Nemerow pollution index

According to Kowalska et al. (2016), PI _Nemerow_ is employed to determine the degree of heavy metal contamination of the soil environment. The formula used to calculate it is as follows:

18 $${PI}_{Nemerow}=\sqrt{\frac{{\left(\frac{1}{N}{\sum }_{1}^{n}PI\right)}^{2}+{\left({PI}_{Max}\right)}^{2}}{2}}$$

*N* stands for total heavy metals, *PI* stands for single pollution index, and *PI *_*max*_ stands for maximum single pollution index for all heavy metals combined.

#### Ecological risk factor

The level of metal toxicity and environmental sensitivity to metal contamination are determined by the ecological risk factor. It is calculated using the equation shown below (Hakanson 1980):

19 $$Er={T}_{i}^{r}\times \frac{{C}_{Sample}}{{C}_{Background}}$$

where *T*_*i*_^*r*^ is the toxic response factor of the specified metal as recommended by Hakanson (1980), and C_Sample_ and C_Background_ are heavy metal concentrations in the sample and background, respectively. The ${T}_{i}^{r}$ values of As, Zn, Mn, and Cr are 10, 1, 1, and 2, respectively (Karuppasamy et al. 2017; Man et al. 2022).

### Analytical technique

#### Gamma spectrometry

A gamma-ray spectrometer was used to measure the activity concentrations of ^210^Pb, ^226^Ra, and ^137^Cs in the sediment samples. The high purity germanium (HPGe) detector (type GEM-50210-P) of the Physics Department of the Faculty of Women for Arts, Science, and Education at Ain Shams University in Egypt had a relative efficiency of almost 50% of the efficiency of the 3″ in 3″ NaI (Tl) crystal. By using the photopeak at 661.6 keV, the specific activity for ^137^Cs was estimated. By using 46.5 keV gamma line energy and after correcting for self-absorption, the specific activity for ^210^Pb was determined. According to Ramos-Lerate et al. (1998), the correction factor was determined by utilizing a ^241^Am (point source) placed over the filled and empty sediment containers. The decay products of the ^226^Ra (^214^Pb at 609, 1120 keV, and ^214^Bi at 295, 352 keV) were used to calculate the specific activity of ^226^Ra. The dry samples were placed in sealed Marinelli beakers and stored for at least 1 month before measurement to ensure radioactive equilibrium between ^226^Ra and ^222^Rn (half-life of 3.8 days) (Lin et al. 2019).

**Fig. 2:**
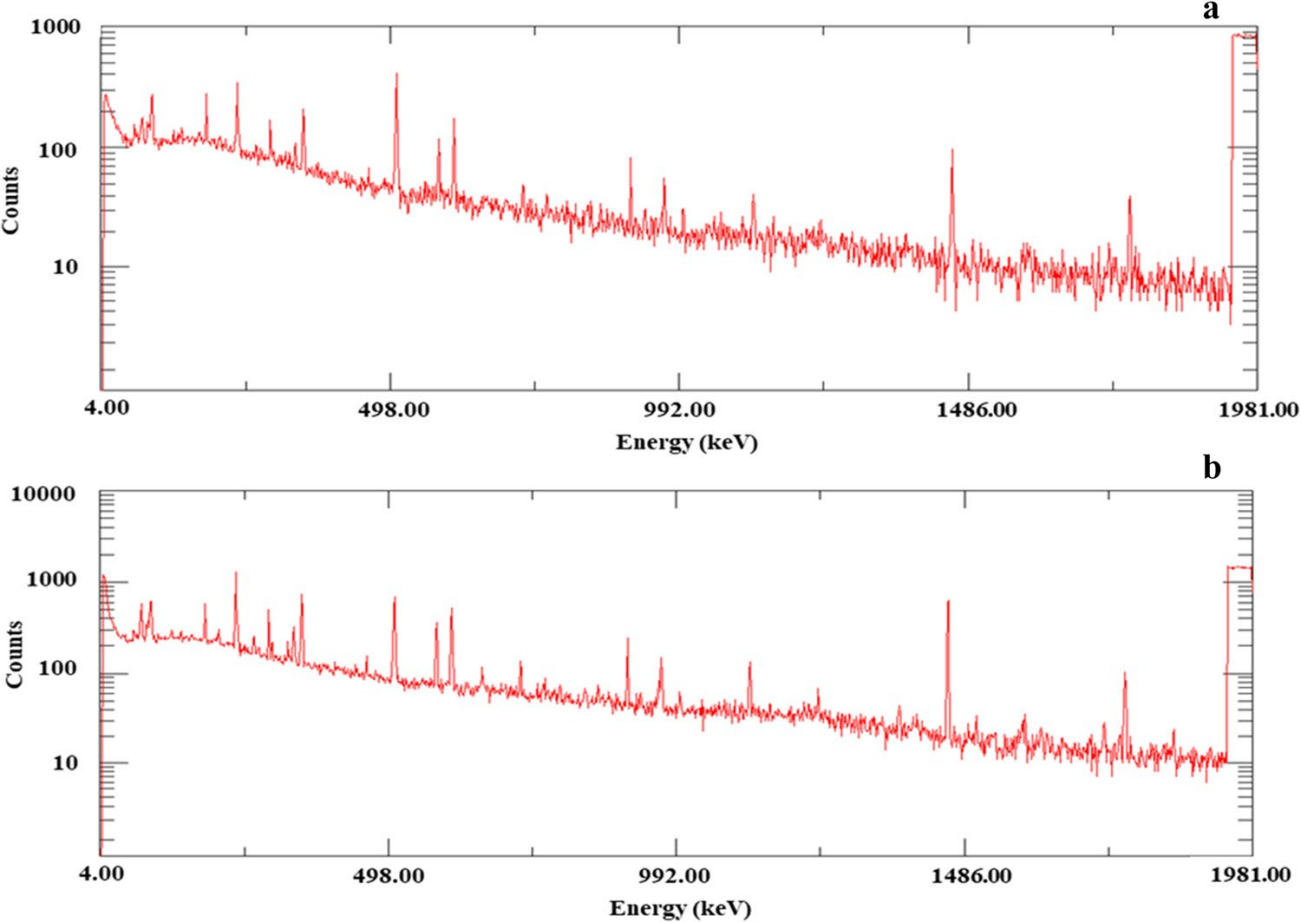
Gamma-ray spectrum of background and sample of core C-2 (4 cm). **a** Background. **b** Sample of core C-2 (4 cm)

The calibration of the detector efficiency was performed by measuring the spectrum of a source emitting γ-rays of precisely known energy, using the IAEA standard sources RGU-1, RGTh-1, RGK-1 (IAEA 1987). In addition, the precision and accuracy of the analysis was estimated using the certified reference materials: IAEA-412 and IAEA-312 (soil) provided by the International Atomic Energy Agency (IAEA). The measured activity concentrations were very close to the reported values of the certified materials, with mean deviations and errors not exceeding 5%. Figure 2-a and 2-b shows a typical gamma spectrum of background and sample of core C-2 (4 cm).

The specific activity (A) (Bq/kg) is calculated as follows (Başkaya et al. 2014; Abbasi 2019):

20 $$A= {~}^{\left({N}_{s}-{N}_{B}\right)}\!\left/ \!{~}_{\left[T\times {I}_{\gamma }\times \varepsilon \times M\right]}\right.$$

where ${N}_{s}$ is the sample’s count rate, ${N}_{B}$ is the background’ count rate, t is the counting time (sec), ${\text{I}}_{\upgamma }$ is the gamma line’s emission probability corresponding to the radionuclide’s peak energy, $\upvarepsilon$ is the spectrometer’s efficiency corresponding to the peak energy, and M is the sample’s weight (kg).

The most important uncertainty sources in our procedure for the determination of the activity concentration of gamma emitters in environmental samples are sample weight, geometry, detector Efficiency, counting statistics, and gamma-ray intensities. The activity concentration uncertainty (U_A_) is calculated by the following equation (Lépy et al. 2015; Rasul et al. 2018; Imam et al. 2024):

21 $${U}_{A}=A\times {\left(\frac{U\left({N}_{s}\right)}{{N}_{s}}+\frac{U\left({N}_{B}\right)}{{N}_{B}}+\frac{U\left({I}_{\gamma }\right)}{{I}_{\gamma }}+\frac{U\left(\varepsilon \right)}{\varepsilon }+\frac{U\left(M\right)}{M}\right)}^{1/2}$$

where $U\left({N}_{s}\right), U\left({N}_{B}\right), U\left({I}_{\gamma }\right), U\left(\varepsilon \right), and U\left(M\right)$ are sample counting, background counting, efficiency, sample mass and gamma line energy uncertainties, respectively. The statistical uncertainty was about 8% in all the energy range.

### Heavy metals determination by INAA

The neutron activation analysis laboratory at the ETRR-2 research reactor in Egypt irradiated 18 sediment core samples to identify the existence of short- and long-lived isotopes, respectively. The sample materials were placed in high-purity polyethylene capsules ranging in size from 100 to 300 mg for both short and long irradiations. Depending on the radionuclides’ half-lives to be measured, there are two different types of irradiations (i.e., irradiations lasting 30 to 60 s for radionuclides with short half-lives and 1 h for radionuclides with long half-lives). Eight elements (Mg, Al, Ca, Ti, V, Na, K, and Mn) were found in our samples after a brief irradiation in which polyethylene capsules containing one sample were transmitted into an irradiation position using a pressurized and pneumatic system. The samples were placed in two groups of aluminum cans, each with two flux monitors (F1 and F2). These cans were packed for prolonged irradiation. Each can hold 9 samples in high-purity polyethylene capsules with a flux monitor. The nuclear parameters of the elements identified through short and long irradiation (keV) are displayed in Table 2.

**Table 2:** Nuclear parameters of the determined elements in short and long irradiation (Glascock 2015)

Element	Activation product	T_1/2_, sec	Gamma-ray used (keV)	Type of radiation
Ti	Ti-51	345.6	320.12	Short
Mg	Mg-27	567.48	843.65, 1014.87	
Mn	Mn-56	9273.6	846.84, 1811.3, 2113.88	
Na	Na-24	53,856	1368.92, 2754.99	
V	V-52	225	1434.5	
Cl	Cl-38	2234.4	1643.73, 2168.24	
Al	Al-28	134.4	1779.52	
K	K-42	44,496	1524.67	
Ca	Ca-49	523.14	3085.36	
Br	Br-82	127,080	554.13, 618.87, 1318.04	Long
As	As-76	94,680	558.87	
Ta	Ta-182	9,886,752	67.5, 1186.39, 1218.81	
Hf	Hf-181	3,662,496	132.8,480.75	
Fe	Fe-59	3,844,800	191.87, 1096.6, 1288.81	
Th	Pa-233	2,332,800	311.01, 339.83	
Cr	Cr-51	2,393,280	319.16	
Cs	Cs-134	64,964,160	603.13, 793.75	
Sc	Sc-46	7,239,456	888.83, 1117.79	
Rb	Rb-86	1,609,632	1074.17	
Zn	Zn-65	21,104,064	1112.84	

**Fig. 3:**
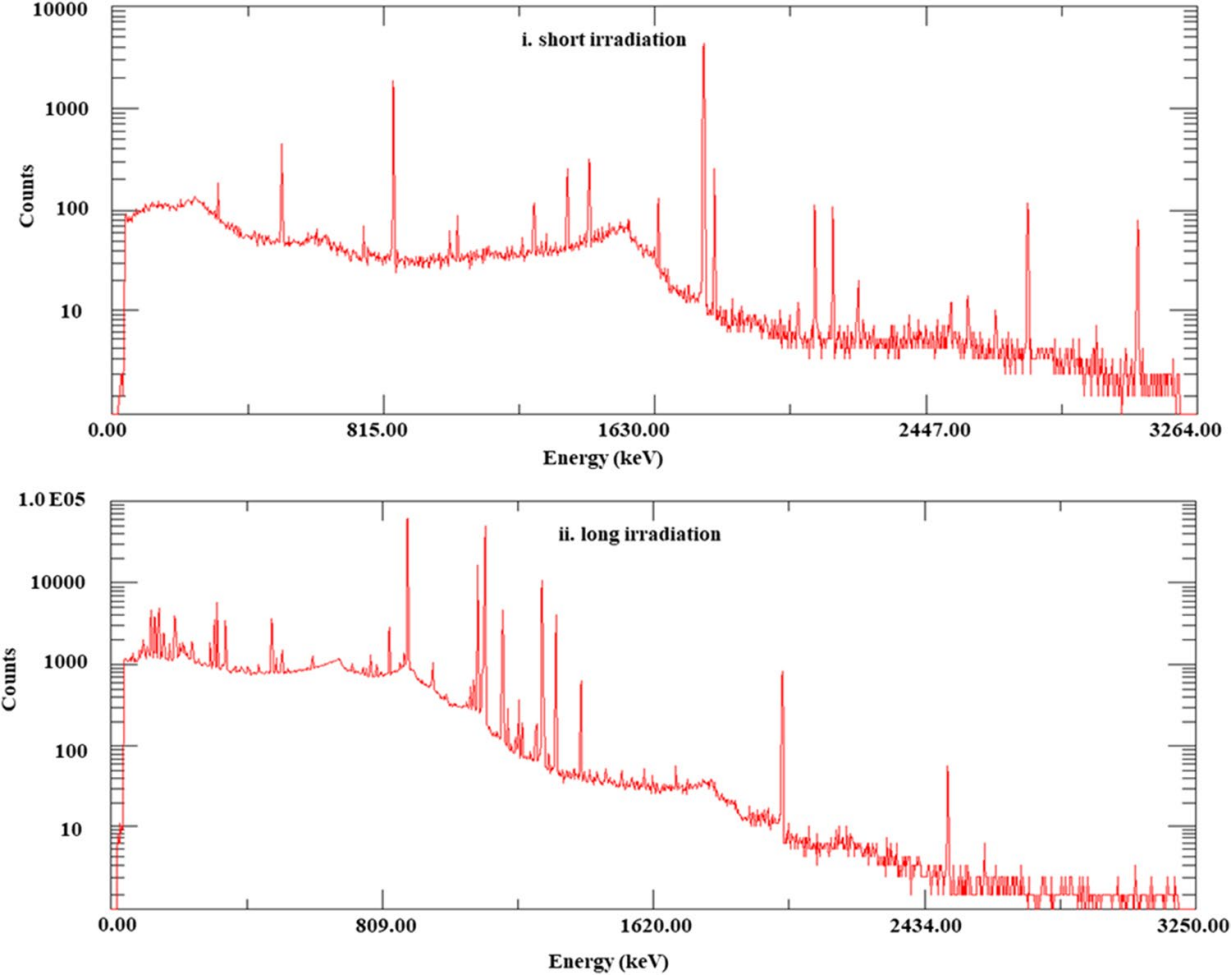
Gamma-ray spectrum of sample core C-2 (4 cm) for short and long irradiation. i Short irradiation; ii Long irradiation

The emitted gamma rays were measured using n-type and p-type HPGe detectors (models GMP-100250-S and GEM-100210-P-Plus, respectively). The gamma ray spectra were analyzed by a gamma vision computer program. Using the ^133^Ba, ^137^Cs, ^60^Co, and ^152^Eu standard point sources from Isotope Products Laboratories, the detector’s energy and efficiency calibrations were carried out. Figure 3-i and ii is an example of the typical gamma-ray spectrum of sample core C-2 (4 cm) after short and long irradiation.

INAA’s relative method was used, specifically k_0_-standardization method (Williams and Antoine 2020). The concentration ${\rho }_{a}$ (ppm) of an element is found from the following equation (De Corte and Simonits 2003).

22 $${\rm P}_{a}= \frac{{\left(\frac{{N}_{p}*correction}{W{t}_{m}SDC}\right)}_{a}}{{\left(\frac{{N}_{p}*correction}{w{t}_{m}SDC}\right)}_{Au}}\times \frac{1}{{K}_{0,Au}\left(a\right)}\times \frac{f+{\varphi }_{0,comp}\left(\alpha \right)}{f+{\varphi }_{0,a}\left(\alpha \right)}\times \frac{{\varepsilon }_{p,comp}}{{\varepsilon }_{p,a}} \times {10}^{6}$$

where “Au” denotes to the gold monitor that has been co-irradiated [^197^Au (n;$\upgamma$)^198^Au, E $\upgamma$ = 411.8 keV], “N_p_” denotes the net number of counts in the full-energy peak, W is the sample weight, w is the gold monitor weight, t_m_ is the measurement time, saturation factor (S=$1-exp\left(-\lambda {t}_{irr}\right), {t}_{irr}$ is irradiation time and $\uplambda$ is the decay constant, decay factor (D = $exp\left(-\lambda {t}_{d}\right)$), ${\text{t}}_{\text{d}}$ is decay time, counting factor (C = $\frac{1-exp\left(-\lambda {t}_{m}\right)}{{t}_{m}}$) and ${\varepsilon }_{p(Comp, a)}$ is the full-energy peak detection efficiency of comparator and element, respectively, f is the thermal to epithermal neutron flux ratio, ${Q}_{0}=\frac{{I}_{0}}{{\sigma }_{0}}$ is resonance integral to 2200 m/s cross-section ratio), and $\alpha$ is the measure for the epithermal neutron flux distribution, approximated by a $\frac{1}{{E}^{\alpha +1}}$ dependence (with $\alpha$ considered to be independent of neutron energy). The uncertainty in the concentration of the element ($\sigma ({\rho }_{a}$)) was obtained by the following relation:

23 $$\sigma ({\rho }_{a})={\rho }_{a}*\sqrt{{{\left(\frac{net\;area\;uncer.}{net\;area}\right)}_{iso.}}^{2}+{{(\sigma }_{\varepsilon ,iso})}^{2}+{{\left(\frac{net\;area\;uncer.}{net\;area}\right)}_{comp}}^{2}+{\left(\frac{\sigma \left(Ko,iso\right)}{100}\right)}^{2}+{\left({\sigma }_{\varepsilon ,comp.}\right)}^{2}}$$

### Quality assurance

The accuracy and precision of the radiometric analysis were estimated by measuring the ^210^Pb values and the ^226^Ra values in the certified reference materials IAEA-410 (radionuclides in bikini atoll sediment), IAEA-312 (radionuclides in soil), and IAEA-314 (radionuclides in stream sediment) that provided by the National Institute of Oceanography and Fisheries, Cairo, Egypt. The activity concentrations obtained for all verified radionuclide values were within 10% of the reported values.

### Statistical analysis

Statistics were used to analyze the analytical data related to the distribution and the relationship between the parameters used in the study. The statistical analysis was carried out using Minitab statistics software and MS Excel (365). Pearson’s correlation matrix was calculated using MS Excel (365) to determine the correlation between elements for the identification of the source of heavy metals. Furthermore, principle component analysis (PCA) is a multivariate statistical technique that uses a linear distribution to minimize the variances in two principal components. It is useful for analyzing multiple variables at once and indicates which parameter in the analysis has greater statistical significance; the *x* component indicates variance that is more significant than the *y* component (Gonçalves et al. 2021).

## Results and discussions

### Radionuclides activity concentration, chronology, and sedimentation rates

**Fig. 4:**
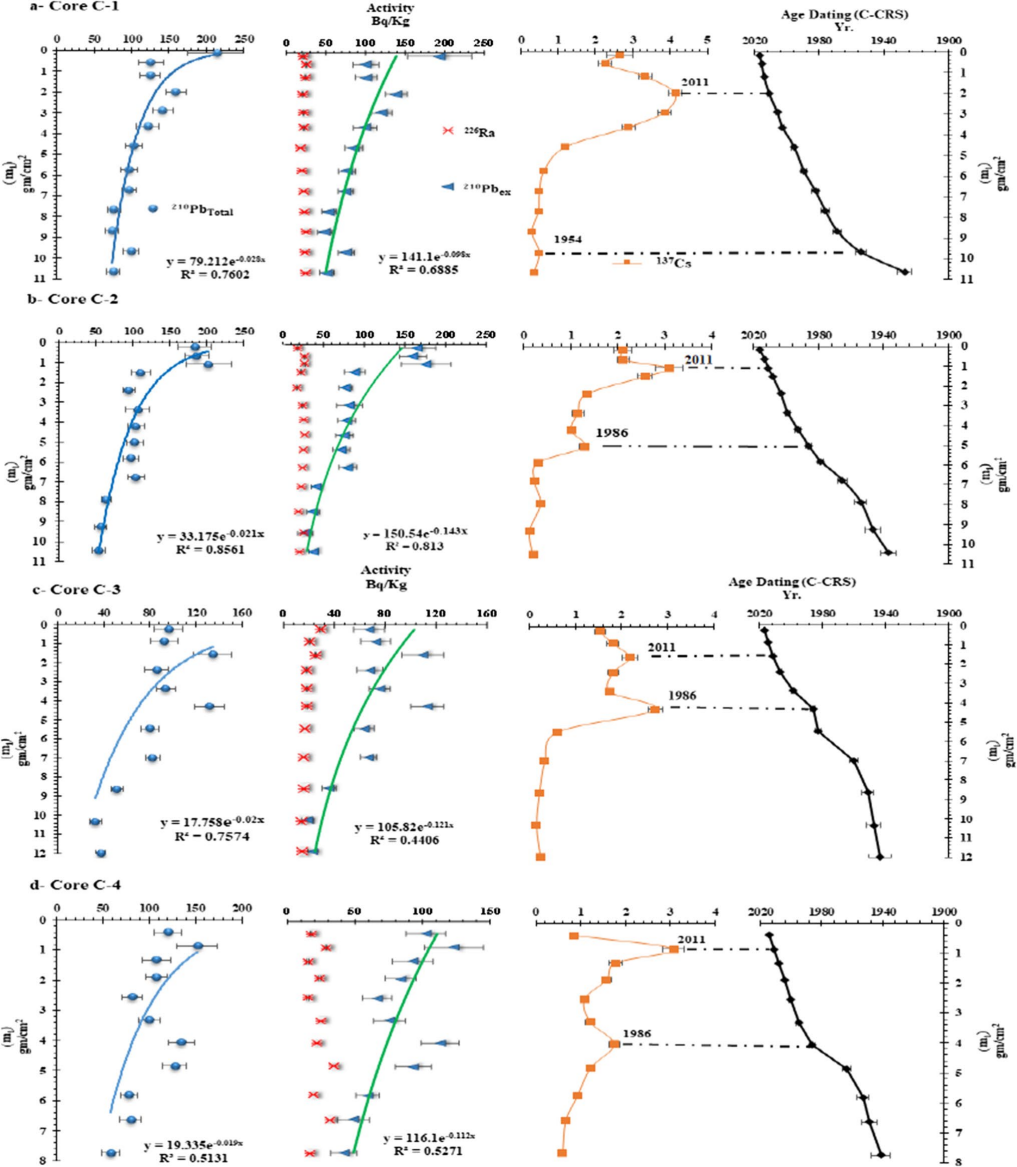
Activity concentration (Bq/Kg) and age-depth model in the core samples (from left to right: ^210^Pb_Total_, ^210^Pb_ex_, ^137^Cs and the C-CRS age model

The distribution of ^210^Pb_total_, ^226^Ra, ^210^Pb_ex_, and ^137^Cs in the sediment cores samples is represented in Table 1S and Fig. 4. The activity concentrations of supported ^226^Ra were practically constant in each core with an average value of 23.37 ± 0.62 Bq/kg, 23.13 ± 0.62 Bq/kg, 18.57 ± 0.44 Bq/kg, and 22.41 ± 0.81 Bq/kg for the core samples C-1, C-2, C-3, and C-4, respectively. The ^210^Pb_ex_ activity concentration can be calculated by subtracting the ^210^Pb_total_ from the supported ^226^Ra. The distribution of ^210^Pb_ex_ activity coincided with that of ^210^Pb_total_ activity due to the relatively constant activity of ^226^Ra. The specific activity of ^210^Pb_ex_ in the core samples typically decline significantly with the cumulative mass depth (Fig. 4). In the sediment cores, the ^210^Pb_ex_ activity profiles can be a good tracer for the dating of marine sediments and the sedimentation rates over the past century. The vertical distributions of the ^210^Pb_ex_ activity profiles were quite irregular, reflecting the biological and/or physical activity of the very extensive aquaculture in the area (Guo et al. 2020). The ^137^Cs radioactivity was measured and varying from 0.30 ± 0.05 to 4.14 ± 0.17 Bq/kg in core C-1, 0.12 ± 0.03 to 3.10 ± 0.29 Bq/kg in core C-2, 0.15 ± 0.02 to 2.18 ± 0.16 Bq/kg in core C-3, and 0.60 ± 0.08 to 3.08 ± 0.24 Bq/kg in core C-4. Since the early 1950s, the^137^Cs has been a part of the environment. There have been two peaks for the ^137^Cs, the first occurring in 1965 as a result of nuclear weapons testing and the second in 1986 as a result of the Chernobyl accident (Appleby 2008). The majority of the sediment cores contain two distinct ^137^Cs peaks, which are peak markers for ^137^Cs from the nuclear accidents at Chernobyl and Fukushima in 1986 and 2011, respectively, except for the sediment core C-1 which had one peak. It is evident that the activity concentration of ^137^Cs is quite low in all sediment cores, which is most likely due to the relatively large losses from the lake by runoff, because of the high solubility of ^137^Cs (Chen et al. 2019). It is possible that the behavior of ^137^Cs in the sediments mirrored that of ^210^Pb_ex_, according to the events recorded in these cores. The chronology of sediment core can be derived from the depth distribution of the ^137^Cs in the sediment profile based on the temporal patterns of the atmospheric fallout from the nuclear tests and accidents.

**Fig. 5:**
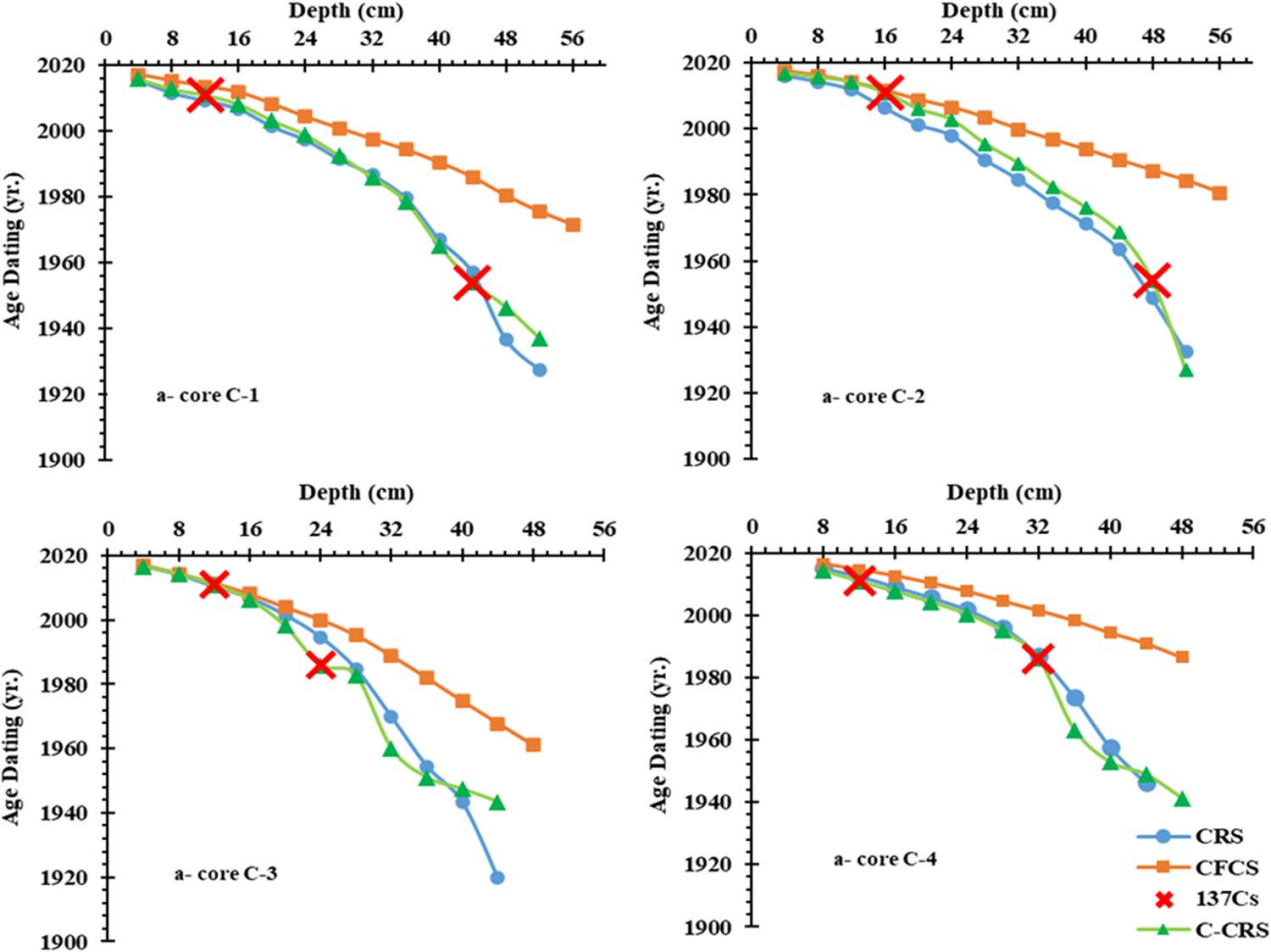
Comparison of the depth-age relation derived from CRS, C-CRS, and CFCS models and the ^137^Cs markers in the core samples

Chronologies were estimated for the cores studied using the CRS, CFCS, and ^137^Cs-corrected CRS (C-CRS) as shown in Fig. 3 and Table 2S. Although these models produced a variety of chronologies, the trends seen in each sediment core were consistent. Compared to other models, the ^137^Cs-corrected CRS model generally produced older ages. The chronologies calculated by the various models revealed a considerable discrepancy in the same core. In the upper layers of C-1, C-2, C-3, and C-4, the chronologies calculated by the CFCS and CRS models showed good agreement. The overestimated and underestimated chronologies in the CFCS model can be explained in part by the rapid urbanization of these coastal areas over the last 50 a, which undoubtedly had an impact on the distribution of ^210^Pb_ex_ in these sediments (Xu et al. 2017). There was agreement between the CFCS and CRS model age estimates for the upper layer of recent sediments (nearly 24 cm) of the four sediment cores, as shown Fig. 5. One of the weaknesses of the CFCS model for these samples is that the CFCS model gives a younger age for the deepest cores than the CRS model. In these samples, the activity concentration of ^137^Cs showed two events in the core samples, except for core C-1 which showed a single peak. In the core C-1, it is obvious that the peak of ^137^Cs at 16 cm represents the event of 2011, the accident of Fukushima, which agrees with the CFCS model (2011.66 ± 0.93). The ages estimated by the CRS model appear to be in good agreement with the chronology based on the ^137^Cs time marker in cores C-2 and C-3 as shown in Table 2S. The small amounts of ^137^Cs concentration have been found in all the sediment cores, which is of interest for the validation of the CRS model. The low concentration of ^137^Cs may be the result of the physical and biological mixing of modern sediments and the diffusion of organisms in pore water (Imam and Salem 2023). The first detected peak of 137Cs was at 32, 24, and 32 cm in cores C-2, C-3, and C-4, which were thought to be related to the Chernobyl accident (AD 1986). In the same context, there are some hypotheses that could be related to the second peak of 137Cs at 12 cm. This peak could be related to global fallout and the nuclear industry, which could be exchanged of the Black Sea with the Mediterranean region (Evangeliou et al. 2009). It may also be due to new 137Cs deposition in the study area from the Fukushima nuclear accident (AD 2011). This is consistent with the distribution of 137Cs found in the eastern Black Sea coast of Turkey (Baltas et al. 2016) and the freshwater Hazar Lake, Turkey (Bilici et al. 2019). Furthermore, the Mediterranean region is already under environmental stress due to heat waves, erratic precipitation, limited water supply, and droughts. Flash floods are therefore relatively common in the Mediterranean and represent one of the most significant natural hazards in the region (Forcing and Region 2014).

**Fig. 6:**
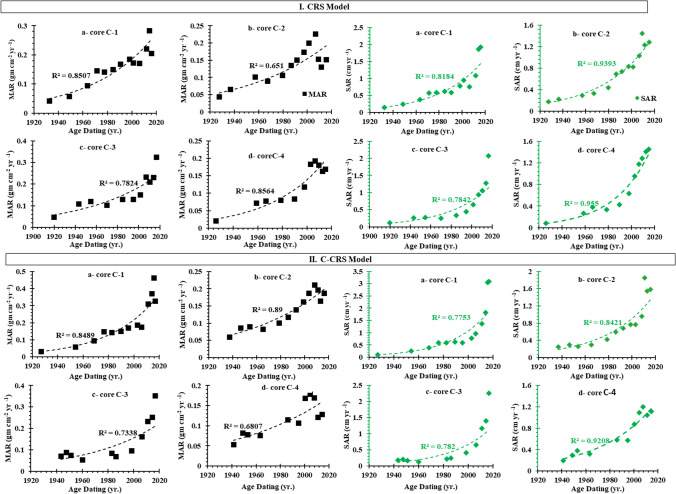
Sediment accumulation rate (SAR, cm year^−1^) and mass accumulation rate (MAR, g cm^−2^ year^−1^) derived from (I) CRS and (II) C-CRS in the cores samples

Table 2S and Fig. 6 show the estimated sedimentation rates based on the CRS and C-CRS models. There are no regular changes in sedimentation rates as a function of core depth, which may be due to variations in atmospheric deposition over time. In the core C-1, the SAR values gradually increased from 1930 to 1980, slightly decreased from 1980 to 1990, and then rapidly increased from 1990 to 2016, as shown in Fig. 6. Obviously, the sedimentation rates increased during 1920 to 2009 and decreased during 2009 to 2016, in the core C-2 (Fig. 6). This may be due to the discharge of wastewater from agricultural and industrial activities. On the contrary, there is successive increase in the sedimentation rates from 1990 to 2017 in core C-3 (Fig. 6). For core C-4, sedimentation rates decrease from 1964 to 1980 as shown in Fig. 6, which is in agreement with Xu et al. (2008). This is because sedimentation on the lower Nile coast in the lagoons via the main river distributaries (Rosetta and Damietta) decreased significantly after the construction of the Aswan High Dam.

It is clear that the sediment cores examined in our study can be divided into two periods, pre and post the High Dam (1964). Our results are compared with previous studies, and we found that the mean sedimentation rate of Burullus Lake before the High Dam (1964) was 0.25 cm a^−1^. This is in agreement with the previous study by Flower et al. (2009) as shown in Table 3. After 1964, the sedimentation rate of Burullus Lagoon was 0.96 cm/a, which is in agreement with Gu et al. (2011). The mean values of sedimentation rates (0.81, 0.96, 0.70, and 0.75 in sediment cores C-1, C-2, C-3, and C-4, respectively) in Burullus Lake were higher than in the other marine environments. The majority of the lagoon area has been lost since the 1980s due to the highway establishing, redevelopment for farming, and growth in urbanization (Hamza 2009), which may be the main cause of the rising sedimentation rates in this lagoon.

**Table 3:** Sedimentation rates and associated dating methods in coastal environments around worldwide

Sites	Sedimentation rates cm yr^−1^	Time spam	Method	Reference
Edku lagoon, Egypt	0.21	Post-1960	^137^Cs	(Chen et al. 2010)
	0.21–0.33	Post-1960	^137^Cs	(Appleby et al. 2001)
Manzala lagoon, Egypt	1.2	Post-Dam	^210^Pb	(Benninger et al. 1998)
	0.2	Post-1960	^137^Cs	(Appleby et al. 2001)
	0.24	Post-1963	^137^Cs	(Appleby et al. 2001)
Burullus lagoon, Egypt	0.3	Post-mid-1960s	^210^Pb	(Flower et al., 2009)
	1.1	Pre-mid-1960s	unknown	(Flower et al., 2009)
	0.14–0.21	Post-1960	^137^Cs	(Appleby et al. 2001)
	0.7	Post-Dam	^210^Pb	(Gu et al. 2011)
Lake Qarun, Western Desert, Egypt	0.45 ± 0.1	––	^210^Pb	(Imam & Ghonamy, 2023)
	0.55		^137^Cs	
San Jose Lagoon, Mexico	0.16–0.51	––	^210^Pb	(Cuellar-Martinez et al. 2017)
Kodaikanal Lake, South India,	0.51 ± 0.14	––	^210^Pb	(Palani et al. 2023)
	0.53 ± 0.08		^137^Cs	
Burullus lagoon, Egypt	0.24^a^	Pre-Dam	^210^Pb	Present study
	0.25^a^	Post-Dam	^137^Cs	
	0.96^a^		^210^Pb	
	1.05^a^		^137^Cs	

^a^Average value

### Vertical distribution of the heavy metals and historical variations of anthropogenic flux

Heavy metal concentration measurements were carried out on three sediment cores C-2, C-3, and C-4, collected from different locations in Burullus Lagoon. The elemental concentrations (μg/g) in the sediment cores of Burullus Lagoon are shown in Table 4. It can be seen that the concentrations of these metals vary considerably in each core. The average concentration of metals in all cores decreased in the following order: Ca > Al > Fe > Mg > Na > Ti >  > Cl > K > Mn > V > Zn > Cr > Rb > Br > Nd > Sc > Th > S > Hf > As > U > Cs. There are some elements that show significant variations in their concentrations such as Mg, Cl, Ca, Ti, V, Na, Zn, Br, Hf, Cr, Mn, Fe, and Ta, while the other metals show values below the detection limit or slightly above the regional background values, according to the abundance of continental shales (Li and Schoonmaker 2014). As the results show, there are three metals with a high degree of variability: Na, Cl, and Mg near El-Boughaz and in the northern parts of the lagoon are due to the seawater intrusion (El-Amier et al. 2016; Badawy et al. 2022). Furthermore, there were high levels of sodium and magnesium near the drains, which may be due to agricultural waste (El-Amier et al. 2016). In the case of the Ca, the average concentration of this element in the sediment cores was about 7–8 times higher than the value of the shale continental abundance. This could be the result of a significant accumulation of shell fragments in the sediments of Burullus Lagoon (El-Amier et al. 2016). Figures 1S, 2S, and 3S show the vertical distributions of metals in the analyzed cores, to demonstrate historical variations in various contaminants linked with sources. In core C-2, the concentration of all elements except Na, Mn, Cl, and Br fluctuated during 2016 to 1990 and increased rapidly from the 1990s to the 1930s. Furthermore, the concentration of Rb, Zn, Th, Cr, Cs, Fe, Sc, and Ta was high in AD 2011 (Fig. 1S); this is probably due to high effluent discharged through drains. Similarly, in core C-3, the highest concentration of most elements was at AD 2017 (Fig. 2S). However, in core C-4, the element content of Rb, Zn, Th, Cr, Cs, Fe, Sc, and Ta decreased during the 1930s to 1940s (Fig. 3S) increased sharply during the 1940s to 1960s and remained slightly constant during the 1960s to AD 2011. The results showed that the average concentrations of the heavy metals were distributed among the cores in the following order: C-2 > C-4 > C-3, indicating high influence of the effluent from the drains. El-Alfy et al. (2017) mentioned that the lagoon receives over 950,000 feddans (approximately 4.1 billion cubic meters per a) of agricultural drainage water, which is approximately 74.3% of the total area of the Nile Delta. Furthermore, after the construction of Aswan High Dam, heavy metals in the Nile sediments increased dramatically (Shetaia et al. 2022). This is evidenced by the upward trend of most of the metals examined in the core samples. This indicates the increased anthropogenic impact.

**Table 4:** Metals concentration (ppm) of the selected cores sediments of the Burullus lagoon

Element	C-2						C-3						Mean value	Shale continental abundance (Li & Schoonmaker 2014)
	Depth (cm)													
	4.00	12.00	20.00	28.00	40.00	52.00	4.00	12.00	20.00	28.00	36.00	44.00		
Na	22,602 ± 1149	18,491 ± 924	14,611 ± 997	11,891 ± 754	12,809 ± 936	8036 ± 489	24,812 ± 1121	19,968 ± 976	30,120 ± 1345	16,021 ± 932	14,358 ± 692	18,087 ± 826	16,899 ± 1290	5900
Mg	24,404 ± 2958	25,104 ± 4178	15,781 ± 3900	24,971 ± 4386	28,789 ± 4463	19,708 ± 3300	22,109 ± 3355	18,036 ± 4009	30,851 ± 4098	17,632 ± 3367	15,197 ± 1169	17,810 ± 2640	21,965 ± 1417	15,000
Al	79,097 ± 2713	69,265 ± 2381	45,305 ± 1573	116,749 ± 3995	139,532 ± 4775	89,565 ± 3077	60,472 ± 2085	53,017 ± 1825	86,868 ± 2980	51,754 ± 1771	49,825 ± 1724	55,106 ± 1886	75,933 ± 7020	88,000
Cl	18,391 ± 1479	15,522 ± 785	8670 ± 716	6162 ± 701	2785 ± 249	2633 ± 429	21,072 ± 940	13,185 ± 874	22,709 ± 1184	6411 ± 513	5118 ± 357	5923 ± 539	9107 ± 1493	180
K	8556 ± 2932	10,720 ± 3831	6676 ± 2085	6911 ± 1268	6949 ± 726	5907 ± 556	9092 ± 3841	6287 ± 1572	4822 ± 2217	5337 ± 1878	7259 ± 1198	10,624.91 ± 1914	6666 ± 515	26,600
Sc	0.40 ± 0.03	40.45 ± 1.16	9.07 ± 0.26	22.05 ± 0.63	32.35 ± 0.93	31.27 ± 0.89	19.60 ± 0.56	13.02 ± 0.37	13.27 ± 0.38	14.87 ± 0.43	13.72 ± 0.39	17.08 ± 0.49	17.62 ± 2.23	13
Ca	197,430 ± 8940	19,9161 ± 8704	40,7337 ± 16,659	37,303 ± 2773	13,583 ± 1640	ND	81,725 ± 4706	89,015 ± 4767	164,919 ± 7558	41,346 ± 2588	19,661 ± 1810	29,746 ± 2489	124,333 ± 26,534	16,000
Ti	11,626 ± 1174	8264 ± 646	5611 ± 978	12,422 ± 1574	11,552 ± 1264	6496 ± 885	9229 ± 1061	47,127 ± 762	11,559 ± 976	5495 ± 614	6949 s ± 750	6613 ± 522	9314 ± 833	4600
Cr	143.60 ± 4.42	197.03 ± 6.08	75.57 ± 2.61	137.85 ± 4.32	193.34 ± 5.96	200.74 ± 6.22	189.24 ± 5.72	123.11 ± 3.90	120.47 ± 3.76	138.23 ± 4.27	130.40 ± 4.05	169.18 ± 4.99	142.93 ± 8.79	90
V	220 ± 21	160 ± 15	125 ± 13	306 ± 30	427 ± 39	263 ± 25	152 ± 15	136 ± 14	232 ± 22	121 ± 11	124 ± 12	130 ± 12	201.40 ± 21.40	130
Mn	2043 ± 324	1773 ± 154	1131 ± 105	729 ± 70	567 ± 57	339 ± 35	952 ± 86	945 ± 85	1500 ± 134	632 ± 60	558 ± 51	638 ± 61	1038 ± 113	850
Fe	54,506 ± 2357	131,185 ± 5672	27,789 ± 1216	56,511 ± 2450	73,043 ± 3158	70,002 ± 3055	63,795 ± 2813	44,150 ± 1931	44,329 ± 1938	55,207 ± 2387	52,754 ± 2278	65,658 ± 2836	58,527 ± 5477	47,200
Zn	138.90 ± 4.87	280.78 ± 10.16	69.37 ± 3.24	227.25 ± 8.42	340.65 ± 11.77	325.83 ± 11.33	217.90 ± 8.12	158.37 ± 6.10	149.89 ± 5.56	166.82 ± 6.08	161.06 ± 6.00	190.88 ± 6.75	195.10 ± 16.40	95
As	2.43 ± 0.56	2.73 ± 0.50	2.58 ± 0.51	3.02 ± 0.37	3.01 ± 0.32	2.20 ± 0.29	3.93 ± 0.72	3.25 ± 0.39	2.51 ± 0.54	3.07 ± 0.37	7.63 ± 0.33	2.10 ± 0.44	2.78 ± 0.32	13
Br	81.45 ± 0.70	75.76 ± 2.86	34.39 ± 1.75	21.50 ± 1.26	15.47 ± 1.08	16.81 ± 0.96	107.76 ± 3.73	59.81 ± 2.14	52.91 ± 2.19	26.76 ± 0.87	19.04 ± 1.11	22.49 ± 1.35	39.42 ± 6.23	20
Rb	55.65 ± 4.79	128.63 ± 10.51	30.37 ± 2.44	65.22 ± 5.16	91.70 ± 6.76	96.15 ± 6.62	71.68 ± 5.51	52.15 ± 4.88	41.84 ± 4.25	56.64 ± 3.92	50.56 ± 4.65	65.52 ± 4.43	61.68 ± 5.85	140
Cs	1.54 ± 0.17	4.41 ± 0.33	1.03 ± 0.14	2.29 ± 0.22	3.48 ± 0.24	3.25 ± 0.24	1.91 ± 0.16	1.42 ± 0.15	1.29 ± 0.10	1.65 ± 0.16	1.22 ± 0.15	1.51 ± 0.17	1.87 ± 0.22	5
Hf	8.00 ± 0.27	13.84 ± 0.48	3.87 ± 0.15	5.16 ± 0.17	7.49 ± 0.27	8.27 ± 0.29	7.26 ± 0.25	5.09 ± 0.19	4.92 ± 0.17	5.24 ± 0.19	5.06 ± 0.18	7.31 ± 0.24	4.65 ± 0.32	5
Ta	2.03 ± 0.12	4.89 ± 0.29	1.14 ± 0.09	1.60 ± 0.11	2.84 ± 0.18	2.67 ± 0.18	2.01 ± 0.10	1.33 ± 0.09	1.32 ± 0.10	1.46 ± 0.10	1.38 ± 0.09	1.59 ± 0.08	1.85 ± 0.22	1.3
Th	6.45 ± 0.20	15.71 ± 0.54	3.57 ± 0.15	8.18 ± 0.25	11.46 ± 0.35	12.15 ± 0.37	6.45 ± 0.21	4.45 ± 0.15	3.98 ± 0.13	4.30 ± 0.14	3.67 ± 0.13	5.25 ± 0.17	6.49 ± 0.81	12

Element	C-4						Mean value	Shale continental abundance (Li & Schoonmaker 2014)
	Depth (cm)							
	12.00	20.00	28.00	36.00	40.00	52.00		
Na	12,343 ± 949	10,368 ± 849	15,940 ± 799	20,028 ± 972	13,760 ± 797	19,939 ± 1230	16,899 ± 1290	5900
Mg	18,012 ± 4271	11,676 ± 3668	26,933 ± 4327	28,308 ± 4294	17,319 ± 2198	32,722 ± 5199	21,965 ± 1417	15,000
Al	46,933 ± 1615	35,194 ± 1208	81,023 ± 2787	103,646 ± 3609	79,543 ± 2739	123,900 ± 4253	75,933 ± 7020	88,000
Cl	8643 ± 737	5808 ± 582	7882 ± 708	4938 ± 466	2549 ± 478	5517 ± 500	9107 ± 1493	180
K	5280 ± 760	3200 ± 421	3308 ± 225	4273 ± 231	8194 ± 2052	6593 ± 595	6666 ± 515	26,600
Sc	12.82 ± 0.37	10.85 ± 0.31	12.02 ± 0.34	13.92 ± 0.40	23.32 ± 0.67	17.03 ± 0.49	17.62 ± 2.23	13
Ca	172,036 ± 8086	220,288 ± 9350	250,287 ± 10,717	147,086 ± 7717	15,977 ± 1732	26,759 ± 2462	124,333 ± 26,534	16,000
Ti	7436 ± 1051	5350 ± 668	10,442 ± 1418	15,685 ± 1562	12,034 ± 1019	16,184 ± 977	9314 ± 833	4600
Cr	118.46 ± 3.76	94.34 ± 3.10	109.44 ± 3.50	116.32 ± 3.74	181.26 ± 5.56	134.08 ± 4.21	142.93 ± 8.79	90
V	113 ± 15	93 ± 9	211 ± 20	269 ± 26	213 ± 21	331 ± 31	201.40 ± 21.40	130
Mn	1088 ± 99	946 ± 87	1794 ± 156	1171 ± 105	648 ± 60	1226 ± 111	1038 ± 113	850
Fe	41,506 ± 1825	35,139 ± 1542	39,913 ± 1750	47,557 ± 2081	85,217 ± 3716	65,219 ± 2822	58,527 ± 5477	47,200
Zn	160.78 ± 6.58	145.15 ± 5.72	151.72 ± 6.41	175.04 ± 7.02	262.78 ± 9.09	189.18 ± 7.46	195.10 ± 16.40	95
As	1.45 ± 0.27	1.52 ± 0.23	1.68 ± 0.17	2.00 ± 0.15	2.95 ± 0.44	1.98 ± 0.27	2.78 ± 0.32	13
Br	48.53 ± 1.81	34.72 ± 1.27	23.35 ± 0.93	18.20 ± 0.77	28.00 ± 1.49	22.54 ± 1.02	39.42 ± 6.23	20
Rb	50.83 ± 4.25	33.96 ± 3.61	33.97 ± 3.10	47.08 ± 4.29	77.17 ± 6.03	61.10 ± 5.72	61.68 ± 5.85	140
Cs	1.16 ± 0.20	0.99 ± 0.13	1.12 ± 0.17	1.32 ± 0.18	2.24 ± 0.21	1.83 ± 0.21	1.87 ± 0.22	5
Hf	4.01 ± 0.14	4.14 ± 0.16	3.97 ± 0.14	4.30 ± 0.15	6.68 ± 0.24	4.82 ± 0.19	4.65 ± 0.32	5
Ta	1.25 ± 0.11	1.15 ± 0.10	1.22 ± 0.10	1.44 ± 0.13	2.37 ± 0.14	1.64 ± 0.12	1.85 ± 0.22	1.3
Th	4.60 ± 0.16	4.29 ± 0.14	4.18 ± 0.14	4.62 ± 0.15	8.01 ± 0.25	5.58 ± 0.19	6.49 ± 0.81	12

*ND* not detected

**Fig. 7:**
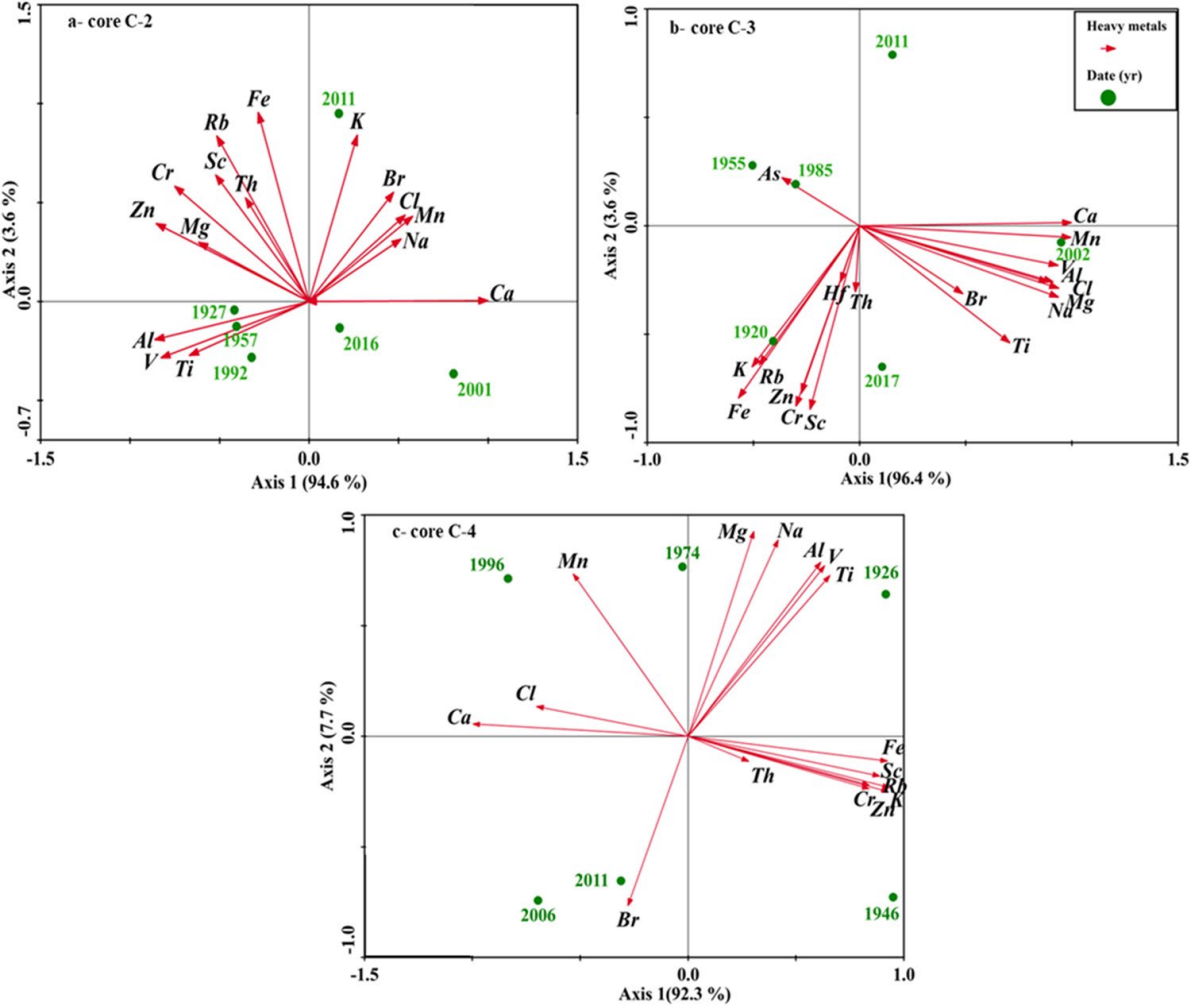
Principle component analysis (PCA) of studied elements with age dating of sediment in three cores

The correlation analysis between the heavy metals in sediment cores samples is presented in Table 5. The strong positive correlations are indicated by a deep red color (correlations close to + 1), while the negative correlations are indicated by a deep blue color (correlations close to − 1). The matrix correlation was created with a significant level of *P* < 0.1 and a confidence level of 0.95%. The strong positive correlation (*r* ≥ 0.7) was observed between Th and Ta, Cs, Rb, Zn, Fe, Cr, Sc; Na—Mn, Br, Cl, K; Ti: V, Mg, and Al (core C-2), while Hf showed positive correlation with Ta, Cs, Rb, Zn, Fe, Cr, Sc, K, and Th; Ti: Mn, V, Cl, Al, Mg, Na, and Br (core C-3). In the same context, there is a significant correlation between Th- Ta, Hf, Cs, Rb, As, Zn, Fe, Cr, Sc, and K; Ti: V, Al, Mg and Na (core C-4). According to the results of Pearson’s correlation coefficient, the substantial positive correlation between metal pairings supported (interpreted) the vertical behavior of these metals for each core. This could be pointing to the same source due to human activities and the weathering of the nearby rocks (Kumar et al. 2019; Hossain et al. 2021). In contrast, Cl showed a negative correlation with other metals (core C-3), and the correlation between Mn and Fe, Zn, As, Br, Rb, Cs, Hf, Ta, and Th (core C-4) indicates a negative or weak correlation, suggesting a different source (El-Alfy et al. 2017). Anthropogenic activities and natural factors influence the distribution of metals in the aquatic environment (Li et al. 2017). The correlations between the investigated elements may also help in identifying the sources of pollution. The results showed that the distribution of heavy metals in sediments can be influenced by changes in human activities. According to the PCA analysis, the sources of metals were grouped into three main categories as shown in Fig. 7. Accordingly, these metals are mainly derived from the same sources of pollution as well as natural weathering (Pan et al. 2016), represented by their association with clay minerals (Khan et al. 2020). This is confirmed by the fact that elements such as Br, Cl, Na, and Mn can be indicators of seawater salinity. Elements such as K, Fe, Th, Rb, Sc, Cr, Mg, Al, and Ti can be indicators of terrigenous origin (Cuellar-Martinez et al. 2017). On the other hand, elements such as Cr, As, V, Zn, and Hf can be derived from terrestrial discharges of wastewater directly discharged from local industrial, aquacultural, and urban areas with metal contamination (Wang et al. 2015).

**Table 5:**
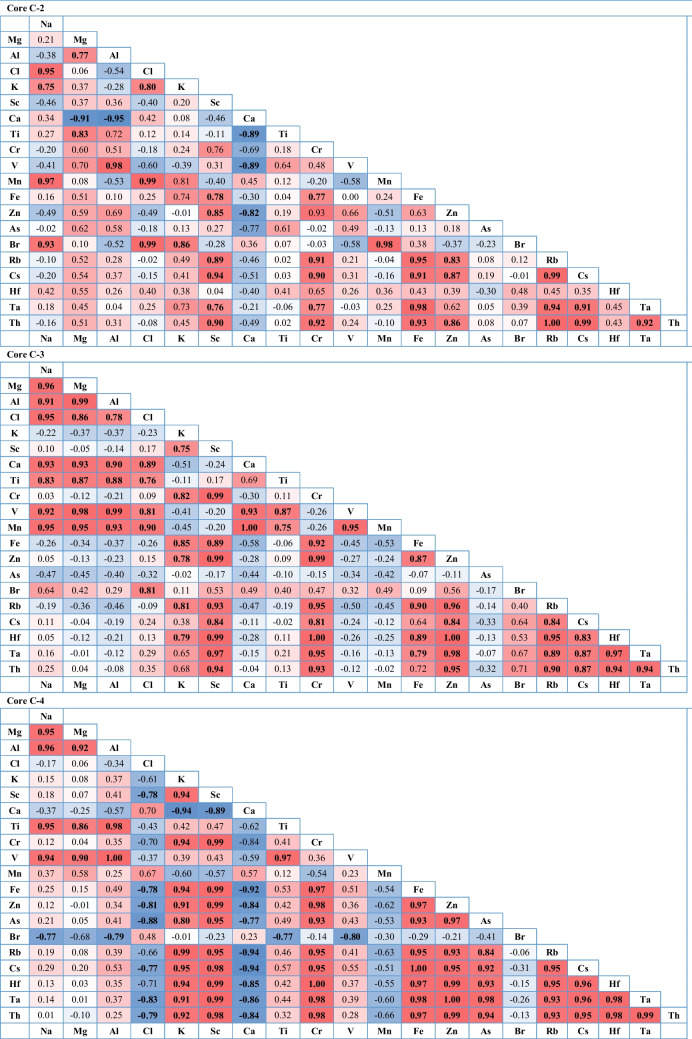
Pearson’s correlation matrix for the element’s concentrations

Bold values: correlation is significant at *P<*0.1

**Fig. 8:**
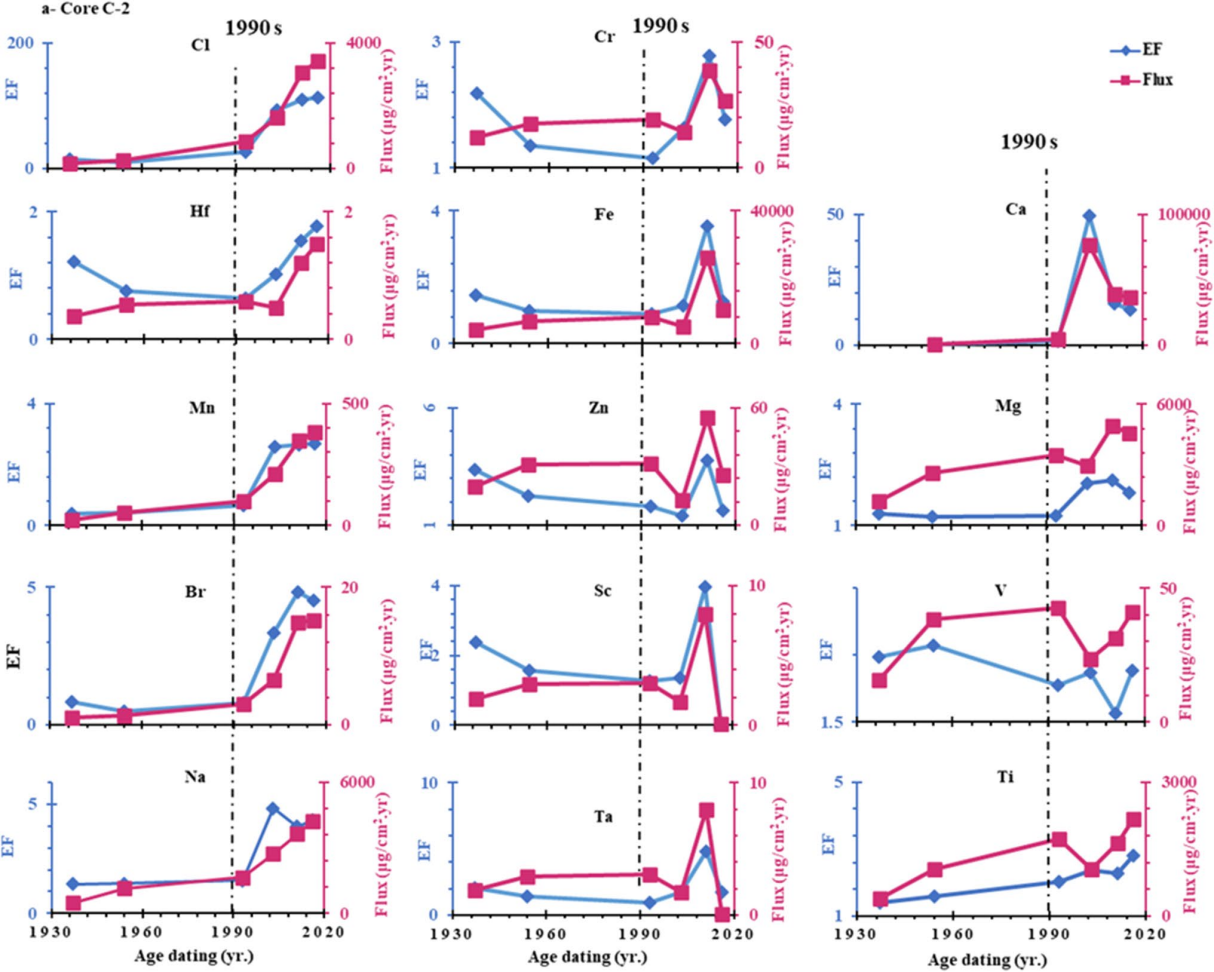

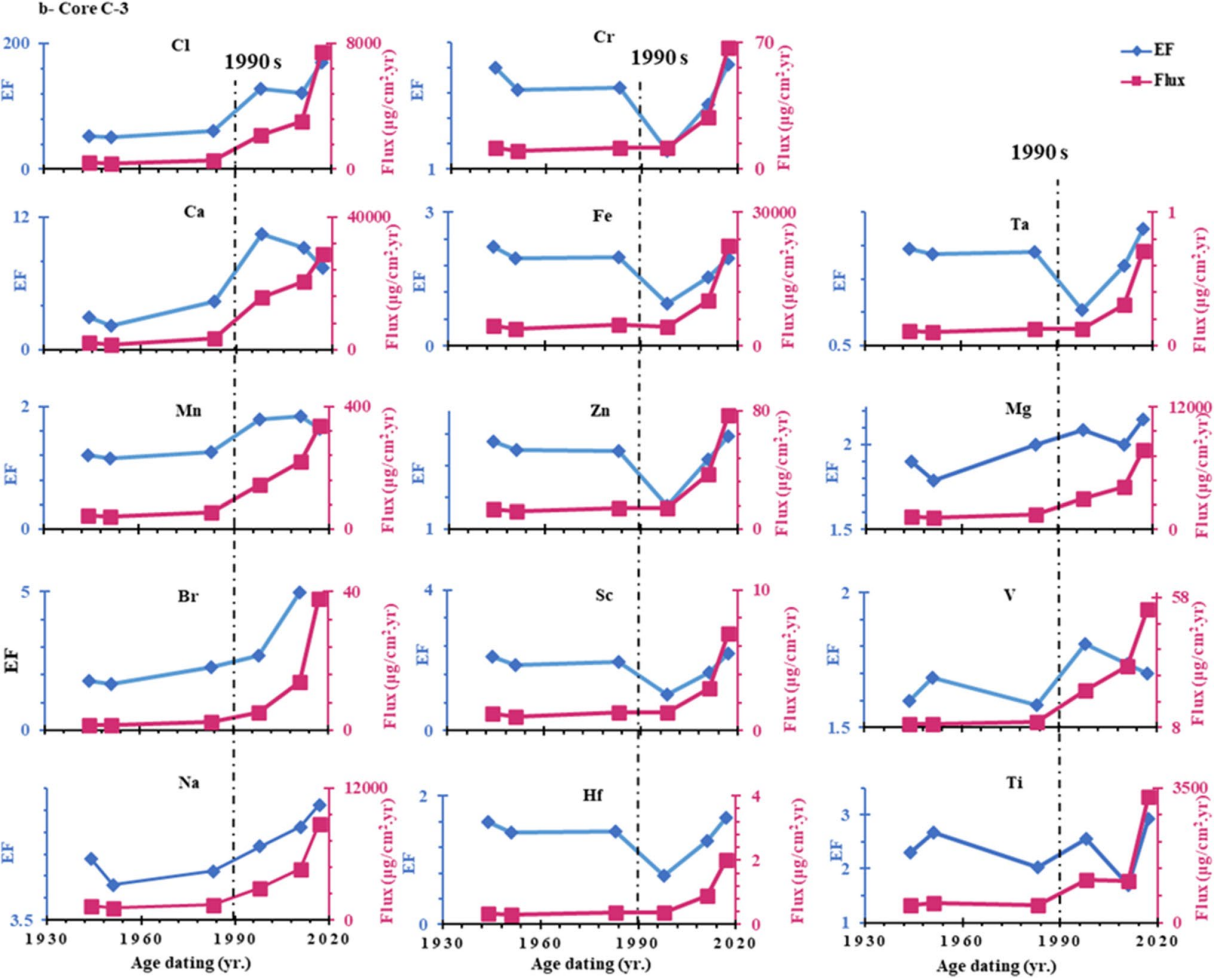

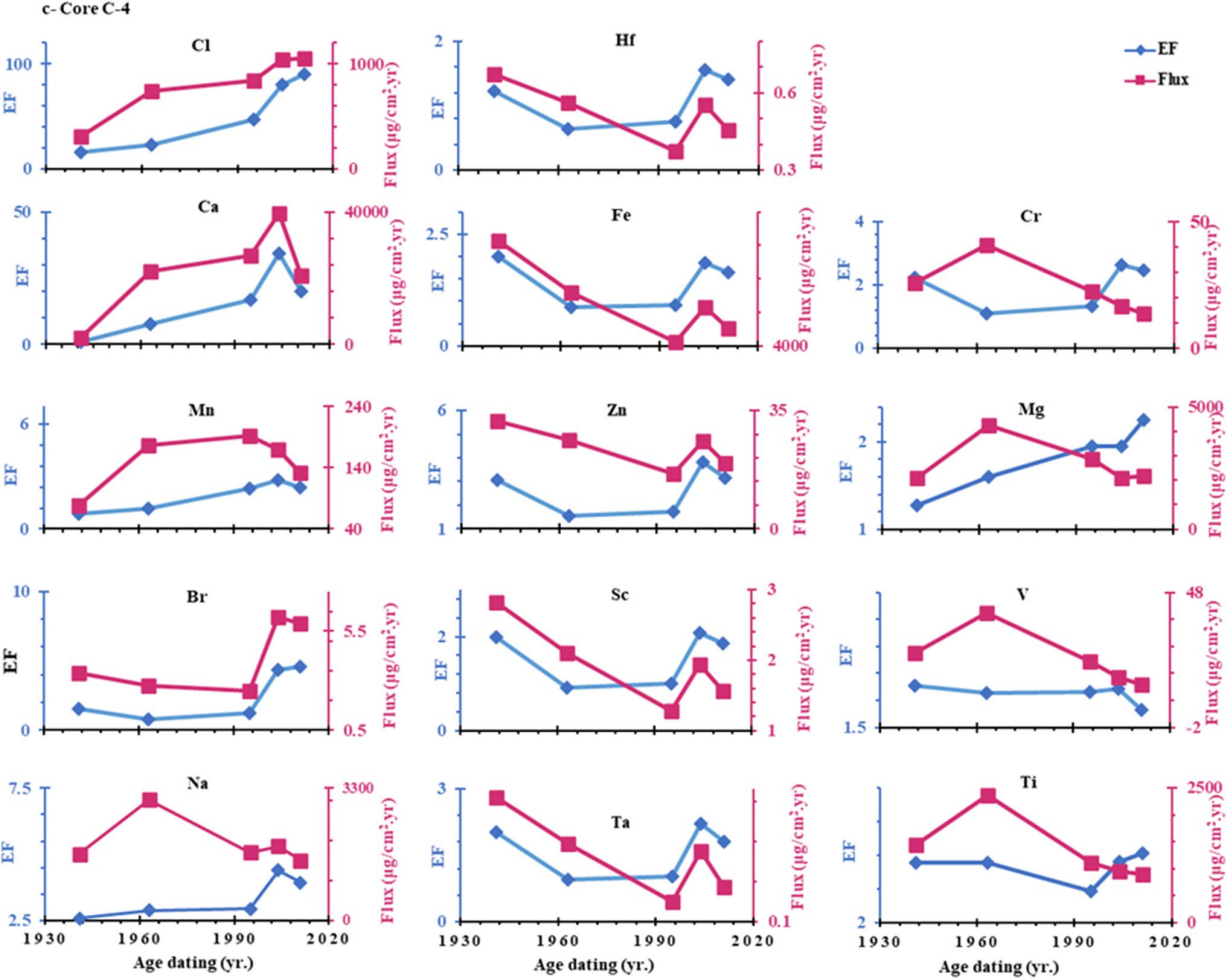
Historical variation of the anthropogenic metal’s enrichment factors and flux in a core C-2, b core C-3, and c core C-4

The concentrations of heavy metals in sediments depend on both the rate of sedimentation at the sampling location and the emission factor of the sources due to the effects of sediment dilution (Wang et al. 2015). Therefore the fluxes reflect anthropogenic contribution much more effectively than their concentrations due to wide variation of sedimentation rates (Bing et al. 2016; Li et al. 2022). The depositional fluxes (μg/cm^2^ a^1^) of Cl, Ca, Na, Br, Zn, Ta, Ti, V, Cr, Sc, Mg, Mn, Fe, Hf were estimated by multiplying the mass accumulation rate g/cm^2^ yr. by anthropogenic concentrations (μg/g) in each section of core (Jha et al. 2002; Ye et al. 2020; Gong et al. 2023). According to Fig. 8, the time-dependent anthropogenic fluxes showed considerable variability and were remarkably similar to the enrichment factor profile found in cores C-2, C-3, and C-4. The mean values of anthropogenic fluxes decreased in order: Ca > Fe > Mg > Na < Cl > Ti > Mn > V > Zn > Cr > Br > Sc > Hf > Ta. The results showed that the sedimentary fluxes of all anthropogenic elements in both cores C-2 and C-3 showed an increasing trend, especially since the 1990s. This could be since several drains, such as 8, 9, El Burullus, and El-Gharbiya, discharge significant amounts of wastewater into the lake along with high concentrations of pesticides and fertilizers, causing severe metal pollution. On the contrary, in core C-4, the depositional fluxes of all elements except Cl, Br and Ca showed a sharp decrease from the 1960s to 2011 AD, as shown in Fig. 6-c. This might be explained by the presence of fresh water supply from the Brimbal canal, which might cause surface sediments to be washed away, and the input of legacy metals under intense soil erosion. We can learn more about past climate change and the effects of human activity on the environment by examining the 100-a records of metals in lagoon sediments (Wang et al. 2019). In comparison with other coastal lagoons, as shown in Table 6, the As concentration in Burullus is lower than in other marine environments. In contrast, the Burullus sediment contains higher levels of Mn, V, Cr, Fe and Zn compared to the former marine environments, indicating anthropogenic activities.

**Table 6:** Comparison of the metal concentrations (μg/g) in the sediment cores of Burullus Lagoon with different countries

Locations	Na	Al	Mn	Mg	Ca	Cl	Ti	As	V	Cr	Fe	Zn	Co	Br	Hf	Ta	Reference
Core C-2	14,740	89,919	1097	23,126	170,963	9027	9329	2.66	250	158	68,839	230	27.57	40.9	5.53	2.52	Present study
Core C-3	20,561	59,507	871	20,273	71,069	12,403	7426	3.75	148.20	145.10	54,316	174.2	29.63	48.10	4.45	1.5	
Core C-4	15,396	78,373	1146	22,495	138,739	5890	11,189	1.93	205	125.6	52,425	180.80	30.25	29.22	3.98	1.51	
Coastal East Malaysia	––	––	––	––	––	––	––	7.09	––	14.57	––	41.78	––	––	––	––	(Ashraf et al. 2018)
Vietnam’s Mong Cai area	––	––	118.52	––	––	––	––	12.49	30.73	20.60	25,400	41.35	5.33	––	––	––	(Loan et al. 2023)
Pearl River Estuary, Southern China	––	––	––	––	––	––	––	––	––	79.77	––	121.77	––	––	––		(Ye et al. 2020)
Sundarbans mangrove forest, Bangladesh (Core 1)	––	––	696	––	––	––	––	81.8	84.0	81.3	42,800	––	15.0	––	––		(Ali et al. 2021)

### The sediment quality indices

The values of heavy metal concentrations do not always represent a system’s true pollution status because this method only considers each metal’s individual severity in terms of biological effects. As a result, the same area could have different pollution states and consequently different conclusions if multiple pollution indices were calculated. In order to thoroughly assess the pollution levels of various heavy metals in the aquatic system of the Burullus Lagoon, EF, PI(_Nemerow_), and E_r_ were used.

### Enrichment factor

Using the enrichment factor (EF), trace element pollution over the previous 100 a was calculated (Li et al. 2022; Man et al. 2022). Table 7 and Fig. 8 show that the average EF values in the examined cores decreased in the following order: Cl > Ca > Na > Br > Zn > Ta > Ti > V > Cr > Sc > Mg > Mn > Fe > Hf. The relatively high EF (EF > 1) values point to anthropogenic pollution, which will require a lot of future attention. The enrichment factors of metals in sediment cores C-2, C-3, and C-4 dramatically increased after mid 1980s as shown in Fig. 8; this may be due to the asynchronous of economic development patterns in this lagoon during this period. This may be due to the increase in economic development patterns in this lagoon during this period which included industrial, residential, fish farm waste and agricultural waste products (Krishnan et al. 2022; Al-Afify et al. 2023), whereas the percentage of gross domestic product (GDP) during the 1980s was 8.9% which was the highest percentage of annual GDP as shown in (https://www.imf.org/external/datamapper).

**Table 7:** Pollution indices (enrichment factors (EF), Nemerow pollution index (PI _Nemerow_), and ecological risk index (E_r_)) in the core samples

Core profile	Depth (cm)	Age Dating (yr.)	EF															PI _Nemerow_	E_r_			
			Na	Mg	Cl	Sc	Ca	Ti	Cr	V	Mn	Fe	Zn	As	Br	Hf	Ta		Cr	Mn	Zn	As
C-2	4.00	2016	4.26	1.81	113.67	0.03	13.73	2.81	1.78	1.88	2.67	1.28	1.63	0.21	4.53	1.78	1.74	2.10	3.19	2.40	1.46	1.87
	12.00	2011	3.98	2.13	109.56	3.95	15.81	2.28	2.78	1.56	2.65	3.53	3.76	0.27	4.81	1.54	4.78	3.11	4.38	2.09	2.96	2.10
	20.00	2003	4.81	2.04	93.56	1.36	49.45	2.37	1.63	1.87	2.58	1.14	1.42	0.39	3.34	1.01	1.70	1.13	1.68	1.33	0.73	1.98
	28.00	1993	1.52	1.25	25.80	1.28	1.76	2.04	1.15	1.77	0.65	0.90	1.80	0.18	0.81	0.65	0.93	2.20	3.06	0.86	2.39	2.32
	44.00	1954	1.37	1.21	9.76	1.57	0.54	1.58	1.35	2.07	0.42	0.98	2.26	0.15	0.49	0.75	1.38	2.72	4.30	0.67	3.59	2.32
	52.00	1937	1.34	1.29	14.37	2.36	ND	1.39	2.19	1.99	0.39	1.46	3.37	0.17	0.83	1.22	2.02	2.07	4.46	0.40	3.43	1.69
Mean Values			2.88	1.62	61.12	1.76	16.26	2.08	1.81	1.86	1.56	1.55	2.37	0.22	2.47	1.16	2.09	2.22	3.51	1.29	2.43	2.05
C-3	4.00	2017	6.12	2.14	170.36	2.19	7.43	2.92	3.06	1.70	1.63	1.97	3.34	0.44	7.84	1.66	2.25	1.82	4.21	1.12	2.29	3.02
	12.00	2011	5.62	2.00	121.58	1.66	9.23	1.70	2.27	1.74	1.85	1.55	2.77	0.41	4.96	1.29	1.70	1.21	2.74	1.11	1.67	2.50
	20.00	1998	5.17	2.08	127.81	1.03	10.44	2.55	1.36	1.81	1.79	0.95	1.60	0.20	2.68	0.76	1.03	2.03	2.68	1.76	1.58	1.93
	28.00	1983	4.62	2.00	60.56	1.94	4.39	2.03	2.61	1.58	1.26	1.99	2.99	0.40	2.28	1.45	1.91	1.33	3.07	0.74	1.76	2.36
	36.00	1951	4.30	1.79	50.22	1.86	2.17	2.67	2.56	1.68	1.16	1.97	2.99	1.04	1.68	1.43	1.87	1.31	2.90	0.66	1.70	5.87
	44.00	1944	4.90	1.90	52.55	2.10	2.97	2.30	3.00	1.60	1.20	2.22	3.21	0.26	1.80	1.59	1.95	1.60	3.76	0.75	2.01	1.62
Mean Values			5.12	1.98	97.18	1.80	6.11	2.36	2.48	1.69	1.48	1.78	2.82	0.46	3.54	1.37	1.79	1.55	3.22	1.02	1.83	2.88
C-4	12.00	2011	3.92	2.25	90.03	1.85	20.16	3.03	2.47	1.63	2.40	1.65	3.17	0.21	4.55	1.41	1.80	1.38	2.63	1.28	1.69	1.12
	20.00	2004	4.39	1.95	80.68	2.09	34.43	2.91	2.62	1.79	2.78	1.86	3.82	0.29	4.34	1.55	2.21	1.04	2.10	1.11	1.53	1.17
	28.00	1995	2.93	1.95	47.56	1.00	16.99	2.47	1.32	1.76	2.29	0.92	1.73	0.14	1.27	0.76	1.02	1.86	2.43	2.11	1.60	1.29
	36.00	1963	2.88	1.60	23.29	0.91	7.81	2.90	1.10	1.76	1.17	0.86	1.56	0.13	0.77	0.64	0.94	2.64	2.58	1.38	1.84	1.54
	44.00	1941	2.58	1.28	15.67	1.98	1.10	2.89	2.23	1.81	0.84	2.00	3.06	0.25	1.55	1.23	2.02	2.20	4.03	0.76	2.77	2.27
	52.00	1938	2.40	1.55	21.77	0.93	1.19	2.50	1.06	1.81	1.02	0.98	1.41	0.11	0.80	0.60	0.90	2.77	2.98	1.44	1.99	1.52
Mean Values			3.19	1.76	46.50	1.46	13.61	2.78	1.80	1.76	1.75	1.38	2.46	0.19	2.21	1.03	1.48	1.98	2.79	1.35	1.90	1.48

### Nemerow pollution index

**Fig. 9:**
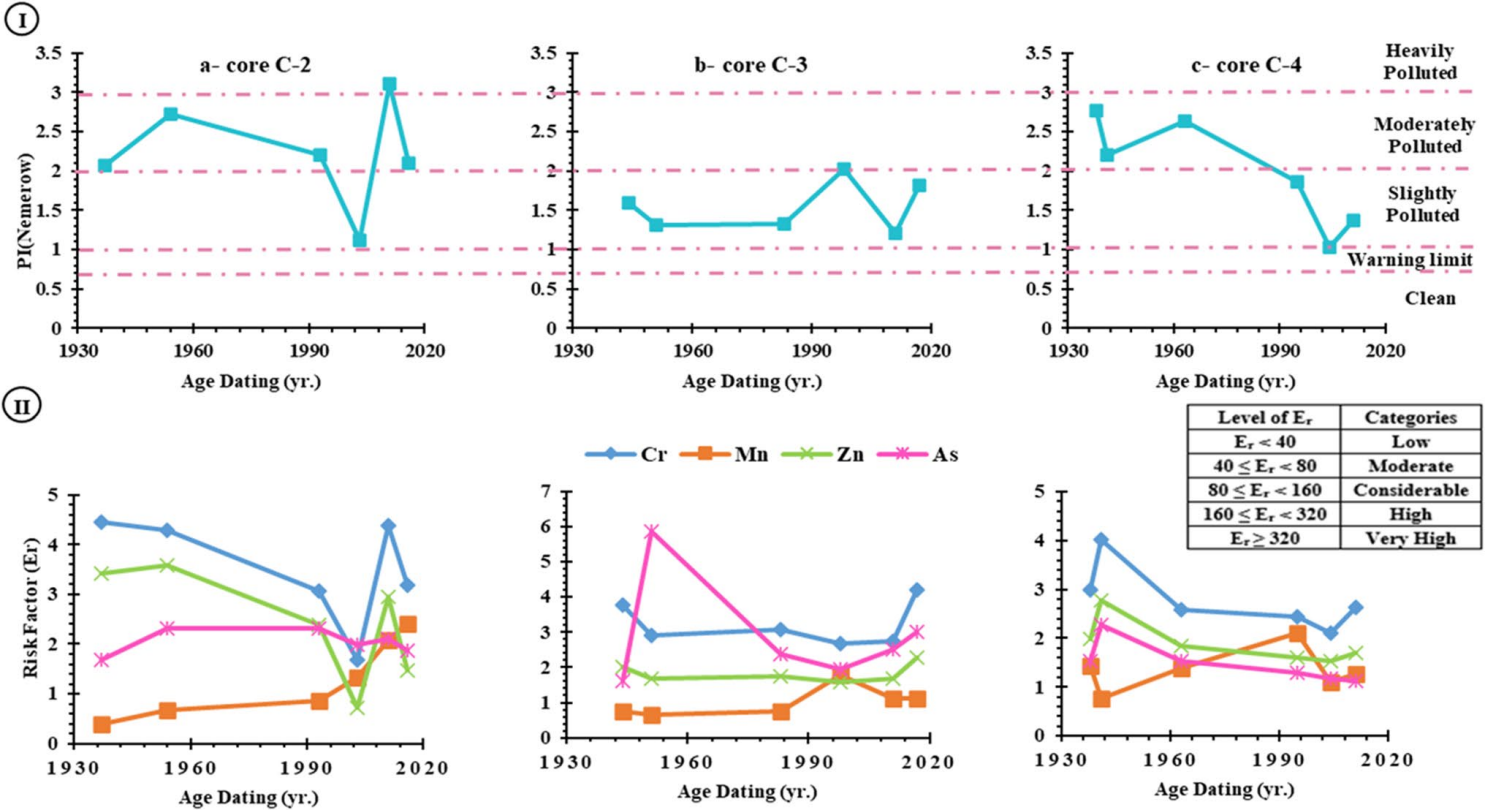
Historical variations of (**I**) PI (Nemerow) and (**II**) risk factor (Er) of the heavy metals in studied core samples

The Burullus lagoon’s sediment quality was evaluated using the Nemerow pollution index. The PI _Nemerow_ values of the heavy metal Zn, Ta, Ti, V, Cr, Sc, Mg, Mn, Fe, and Hf showed that the sediments of core C-2 were moderately polluted from 1937 to 1993, with values generally between 2 and 3 as shown in Fig. 9-I. In addition, PI _Nemerow_ was between 1 and 2 in 2003, indicating light pollution, while in 2011, values were between 2 and 3, indicating heavy pollution (Fig. 9-I). In the same context, all the values of PI _Nemerow_ were between 1 and 2, indicating that the sediments of core C-3 were severely polluted from 1944 to 2017 (Fig. 9-I). On the other hand, for core C-4, between 1938 and 1963, the values of PI _Nemerow_ were between 2 and 3, indicating moderate pollution, while from 1995 to 2011, the values of PI _Nemerow_ were slightly polluted, as shown in Fig. 9-I. The PI _Nemerow_ values ranged from 1.13 to 3.11 with a mean value 2.22 for the core C-2, 1.21 to 2.03 with a mean value 1.55 for core C-3, and from 1.04 to 2.77 with mean value 1.98 for core C-4 as demonstrated in Table 7. The vertical distribution of PI _Nemerow_ showed that the values increased in the direction of the drains and the mean values arranged the studied cores in the following order C-2 > C-4 > C-3 and were slightly polluted. According to the PI _Nemerow_ category interpretation suggested by Kowalska et al. (2018), this may be attributed to the high content of heavy metals in cores C-2 and C-4.

### Ecological risk factor

The ecological risk factor (Er), which is calculated for Co, Cr, Mn, As, and Zn, is an individual pollution indicator that assesses the possible risk posed by a single element. Except for core C-3, the calculated Er values for each heavy metals in the examined cores are in the following order: Cr > Zn > As > Mn, as shown in Table 7 and Fig. 9-II. The Er values show that there was little contamination in the sediments of cores C-2, C-3, and C-4 during the investigation period.

### Conclusions and recommendations:

Discharges of agricultural, industrial, and domestic effluents from several drains have caused changes in the sedimentological regime, degradation of sediment quality, and increased sedimentation rates in Burullus Lagoon. Therefore, this study highlights the use of ^210^Pb dating models to establish an accurate chronological framework for recent sediments in order to understand historical climate changes and the impact of human activities on the ecology of Burullus Lagoon. Two main models (CRS and CFCS) were tested to determine these chronologies. The ^210^Pbex and ^137^Cs activity profiles were used to calculate the sedimentation rates studied. The results show that the constant rate of supply (CRS) model performed best and appears to be in good agreement with the chronology based on the ^137^Cs technique in the investigated cores. The age estimates from the CFCS and CRS models agreed for the recent sediments in the uppermost layer of the four cores. As a result of our analysis, possible explanations for the ^137^Cs peaks include the nuclear industry, global fallout, possible exchange of the Black Sea with the Mediterranean region, and the Fukushima (2011 AD) and Chernobyl (1986 AD) accidents. The mean values of sedimentation rates (0.81, 0.96, 0.70 and 0.75 in sediment cores C-1, C-2, C-3, and C-4, respectively) in the Burullus lagoon were higher than in the other marine environments. This could be due to the construction of new roads, urbanization, and agricultural reclamation, which have consumed most of the lagoon area since the 1980s, in line with the annual percentage of GDP during this period. It can also be explained by the different dynamic geophysical and/or hydrological conditions that each site has, which control the sedimentation process. This study explains how drainage water discharged into Burullus Lagoon, carrying silt loads, has historically influenced sedimentation rates.

Due to its high sensitivity and minimal sample handling requirements, NAA has been used to identify contamination standards and to detect trace impurities, whereas the INAA technique has a wide range of potential applications because it can simultaneously and non-destructively provide accurate data on a large number of elements at the nanogram level. The main disadvantage of using NAA is that the irradiated sample remains radioactive for many years after the initial analysis, requiring special handling and disposal techniques for low to intermediate level radioactive material. In the same context, the XRF method is also non-destructive, but it requires the analysis of a large amount of sample (usually more than one gram). Furthermore, the difficulty of identification of elements lighter than sodium and the difficulty of discrimination between isotopes of the same element or ions of the same element in different valence states are some of the disadvantages of the XRF method. Therefore, the k0-INAA technique has been successfully applied for a sensitive and reliable multi-element analysis for the assessment of environmental changes in sediments and the impact of anthropogenic activities during 100 a in the Burullus lagoon.

The results show that elements such as Mg, Cl, Ca, Ti, V, Na, Zn, Br, Hf, Cr, Mn, Fe, and Ta have concentrations above background in the continental shales. Core C-2 at El-Boughaz has higher concentrations of Na, Cl, and Mg than the other cores C-3 and C-4; this could be due to seawater intrusion. The mean concentration of Ca in the sediment cores was about 7–8 times higher than the continental abundance of the shale. A possible explanation for this could be a significant accumulation of shell fragments in the sediments of the Burullus lagoon. As a result of the results, it can be concluded that there are different sources in the Burullus lagoon, based on the variable vertical distribution of elements in the core samples. Strong positive correlations between most of the elements studied indicate similar sources, most likely as a result of human activity and weathering of the surrounding rocks. The information presented here is the first attempt to use sediment cores from Burullus Lagoon to estimate the fluxes of different elements in the Nile Delta. The observed increase in fluxes of anthropogenic elements in cores C-2 and C-3 since the 1990s is mainly due to run-off, overfishing, increased fish farming, and sewage discharge. These anthropogenic elements include Br, Ca, Cl, Cr, Fe, Na, Sc, Ti, Hf, V, Fe, Mg, Mn, and Zn. The input of legacy metals under severe soil erosion and the availability of fresh water from the Brimbal Canal, which may have caused washing of surface sediments, are probably responsible for the sharp decrease in fluxes of some elements in core C-4 from the 1960s to 2011 AD. The asynchronous economic development patterns in this lagoon may be the cause of the dramatic increase in metal enrichment factors in sediment cores C-2, C-3, and C-4 after the mid-1980s.

According to the results, anthropogenic activities may have an impact on Burullus Lake. Monitoring is necessary to give coastal zone managers and decision-makers the knowledge they need to take serious protective measures for this lake, which is one of Egypt’s most valuable economic resources. Pre-treatment of wastewater prior to discharge into lakes, control of additional pollutants from chemical pesticides and fertilizers used on agricultural crops, and lake water replenishment with seawater should all be included in these measures.

## Supplementary Information

Below is the link to the electronic supplementary material.

Supplementary file1 (DOCX 213 KB)

## Data Availability

The datasets and materials used during the current study are available from the corresponding author on reasonable request. All data generated or analyzed during this study are included in this published article.
