# Correction to: Historical trends of heavy metals applying radio‑dating and neutron activation analysis (NAA) in sediment cores, Burullus Lagoon, Egypt

**DOI:** 10.1007/s11356-024-34102-2

**Published:** 2024-07-02

**Authors:** Alia Ghanem, Afaf Nada, Hosnia Abu‑Zeid, Waiel E. Madcour, Said A. Shetaia, Noha Imam

**Affiliations:** 1 Physics Department, Faculty of Women for Arts, Science & Education, Ain Shams University, Cairo, Egypt; 2 Radiation Protection Department, Nuclear Research Center, Egyptian Atomic Energy Authority, Cairo, Egypt; 3 Geology Department, Faculty of Science, Al-Azhar University, Cairo, Egypt; 4 Physics & Geology Lab, National Institute of Oceanography and Fisheries, Cairo, Egypt

**Correction to: Environmental Science and Pollution Research**

10.1007/s11356-024-33761-5

The 4th author name is missing the middle initial. It should be Said A. Shetaia.

The Original article has been corrected.

