# Supplementary material for: Historical trends of heavy metals applying radio-dating and neutron activation analysis (NAA) in sediment cores, Burullus Lagoon, Egypt
Source: Environ Sci Pollut Res Int. 2024 Jun 13;31(31):43633–58. doi: 10.1007/s11356-024-33761-5 (PMC11512863; doi:10.1007/s11356-024-33761-5)
Supplement: Supplementary file 1 — Supplementary file1 (DOCX 213 KB) [file 11356_2024_33761_MOESM1_ESM.docx]

Historical trends of heavy metals applying radio-dating and Neutron Activation Analysis (NAA) in sediment cores, Burullus Lagoon, Egypt.

A. Ghanem ^a^, A. Nada ^a^, Hosnia M. Abu-zeid ^a^, Waiel E. Madcour ^b^, Said A. Shetaia ^c^, N. Imam ^d, *^

^a^ Physics Department, Faculty of Women for Arts, Science & Education, Ain Shams University, Cairo, Egypt

^b^ Radiation Protection department, Nuclear Research Center, Egyptian Atomic Energy Authority, Cairo, Egypt

^c^ Geology Department, Faculty of Science, Al-Azhar university, Cairo, Egypt

^d^ Physics & Geology Lab, National Institute of Oceanography and Fisheries, Cairo, Egypt

**Journal of Environmental Science and Pollution Research (JESPR)**

***Corresponding author**:

-Dr. Noha Abdalla Mohamed Imam,

E-mail: [noha_imam115@hotmail.com](mailto:noha_imam115@hotmail.com)

na.imam@niof.sci.eg

**Table 1S: The activity concentration of ^210^Pb_Total_, ^226^Ra, ^210^Pb_ex_ and ^137^Cs in core sediment samples.**

| Core profile | Depth  cm | Mass depth  g cm^-1^ | ^210^Pb_Total_  Bq/Kg | ^226^Ra  Bq/Kg | ^210^Pb_ex_  Bq/Kg | ^137^Cs  Bq/Kg |
| --- | --- | --- | --- | --- | --- | --- |
| C-1 | **4.00** | **0.16±0.003** | **214.86±40.61** | **21.05±1.03** | **193.81±40.62** | **2.66±0.35** |
|  | **8.00** | **0.58±0.01** | **125.84±16.38** | **25.21±0.79** | **100.63±16.62** | **2.27±0.17** |
|  | **12.00** | **1.19±0.02** | **125.06±13.81** | **24.00±0.67** | **101.05±13.62** | **3.33±0.17** |
|  | **16.00** | **2.00±0.04** | **159.42±13.62** | **20.52±0.55** | **138.90±13.62** | **4.14±0.17** |
|  | **20.00** | **2.91±0.06** | **142.09±13.25** | **21.56±0.57** | **120.53±13.62** | **3.86±0.17** |
|  | **24.00** | **3.64±0.07** | **121.62±14.86** | **22.03±0.67** | **99.59±14.62** | **2.88±0.18** |
|  | **28.00** | **4.60±0.09** | **103.12±11.32** | **17.83±0.54** | **85.29±11.62** | **1.19±0.09** |
|  | **32.00** | **5.74±0.11** | **96.30±10.83** | **19.66±0.52** | **76.64±10.62** | **0.63±0.07** |
|  | **36.00** | **6.71±0.13** | **96.81±9.59** | **21.92±0.65** | **74.89±9.62** | **0.50±0.05** |
|  | **40.00** | **7.68±0.15** | **76.60±9.03** | **22.73±0.45** | **53.87±9.62** | **0.48±0.05** |
|  | **44.00** | **8.70±0.17** | **73.61±9.27** | **24.87±0.62** | **48.75±9.62** | **0.30±0.05** |
|  | **48.00** | **9.69±0.19** | **99.15±10.39** | **23.50±0.59** | **75.65±10.62** | **0.50±0.06** |
|  | **52.00** | **10.65±0.21** | **75.61±8.91** | **24.32±0.57** | **51.29±8.62** | **0.36±0.05** |
|  | **56.00** | **0.191±0.004** | **73.06±7.27** | **23.93±0.48** | **49.14±7.62** | **0.32±0.04** |
| C-2 | **4.00** | **0.664±0.013** | **183.48±22.42** | **17.92±0.58** | **165.56±5.11** | **2.11±0.19** |
|  | **8.00** | **1.088±0.022** | **186.55±16.69** | **26.51±0.69** | **160.04±5.08** | **2.12±0.14** |
|  | **12.00** | **1.513±0.030** | **202.57±30.54** | **26.37±1.05** | **176.20±5.17** | **3.10±0.29** |
|  | **16.00** | **2.394±0.048** | **110.71±12.78** | **22.28±0.61** | **88.43±4.48** | **2.59±0.15** |
|  | **20.00** | **3.365±0.067** | **94.14±8.01** | **17.10±0.39** | **77.04±4.34** | **1.34±0.07** |
|  | **24.00** | **4.206±0.084** | **106.09±15.81** | **24.21±0.83** | **81.88±4.41** | **1.15±0.13** |
|  | **28.00** | **5.025±0.101** | **104.09±11.03** | **25.61±0.62** | **78.48±4.36** | **1.01±0.08** |
|  | **32.00** | **5.813±0.116** | **102.51±10.87** | **26.77±0.62** | **75.74±4.33** | **1.29±0.09** |
|  | **36.00** | **6.773±0.135** | **97.08±10.53** | **25.09±0.62** | **71.98±4.28** | **0.31±0.05** |
|  | **40.00** | **7.871±0.157** | **104.13±11.04** | **24.06±0.63** | **80.07±4.38** | **0.23±0.04** |
|  | **44.00** | **9.255±0.185** | **63.64±6.27** | **22.49±0.44** | **41.15±3.72** | **0.36±0.04** |
|  | **48.00** | **10.436±0.209** | **56.25±7.65** | **19.17±0.53** | **37.09±3.61** | **0.12±0.03** |
|  | **52.00** | **0.24±0.01** | **53.33±9.28** | **25.82±0.72** | **27.51±3.31** | **0.19±0.04** |
|  | **56.00** | **0.87±0.02** | **58.37±5.75** | **20.41±0.39** | **37.96±3.64** | **0.27±0.03** |
| C-3 | **4.00** | **1.59±0.03** | **96.24±12.12** | **29.06±0.72** | **67.18±12.15** | **1.53±0.12** |
|  | **8.00** | **2.38±0.05** | **92.85±11.70** | **20.37±0.58** | **72.48±11.71** | **1.81±0.13** |
|  | **12.00** | **3.38±0.07** | **134.58±16.44** | **25.10±0.46** | **109.48±16.45** | **2.18±0.16** |
|  | **16.00** | **4.30±0.09** | **85.95±10.13** | **18.10±0.51** | **67.84±10.14** | **1.82±0.11** |
|  | **20.00** | **5.46±0.11** | **93.95±8.30** | **18.31±0.42** | **75.63±8.31** | **1.76±0.09** |
|  | **24.00** | **6.98±0.14** | **131.39±12.88** | **18.31±0.51** | **113.08±12.89** | **2.74±0.14** |
|  | **28.00** | **8.63±0.17** | **80.21±7.87** | **16.78±0.41** | **63.43±7.88** | **0.61±0.05** |
|  | **32.00** | **10.34±0.21** | **82.47±6.32** | **15.72±0.32** | **66.75±6.33** | **0.34±0.03** |
|  | **36.00** | **11.99±0.24** | **51.72±5.49** | **15.97±0.35** | **35.75±5.40** | **0.22±0.03** |
|  | **40.00** | **0.41±0.01** | **33.21±4.90** | **14.13±0.36** | **19.08±4.91** | **0.16±0.03** |
|  | **Continue Table 1S:** | | | | | |
|  | **44.00** | **0.87±0.02** | **37.55±4.00** | **14.54±0.29** | **23.01±4.01** | **0.24±0.02** |
|  | **48.00** | **1.34±0.03** | **39.36±5.04** | **16.51±0.39** | **22.84±5.05** | **0.15±0.02** |
| C-4 | **8.00** | **1.90±0.04** | **119.82±14.75** | **17.34±0.57** | **102.48±4.63** | **0.86±0.10** |
|  | **12.00** | **2.56±0.05** | **151.80±21.91** | **28.51±0.57** | **123.29±4.81** | **3.08±0.24** |
|  | **16.00** | **3.32±0.07** | **107.85±15.10** | **15.12±0.78** | **92.72±4.53** | **1.78±0.15** |
|  | **20.00** | **4.07±0.08** | **107.41±11.58** | **23.53±0.81** | **83.87±4.43** | **1.58±0.11** |
|  | **24.00** | **4.86±0.10** | **81.48±10.79** | **15.09±0.65** | **66.39±4.20** | **1.08±0.10** |
|  | **28.00** | **5.82±0.12** | **100.02±11.79** | **24.56±0.91** | **75.47±4.32** | **1.21±0.10** |
|  | **32.00** | **6.64±0.13** | **134.68±13.97** | **21.55±1.02** | **113.14±4.73** | **1.75±0.12** |
|  | **36.00** | **7.74±0.15** | **127.28±13.27** | **34.20±1.08** | **93.08±4.53** | **1.22±0.10** |
|  | **40.00** | **0.16±0.003** | **78.32±8.65** | **19.06±0.70** | **59.26±4.08** | **0.92±0.07** |
|  | **44.00** | **0.58±0.01** | **80.04±11.80** | **31.29±1.07** | **48.75±3.89** | **0.66±0.08** |
|  | **48.00** | **1.19±0.02** | **57.99±9.66** | **16.31±0.75** | **41.68±3.73** | **0.60±0.08** |

**Table 2S: Calendar dates (yrs.) and sedimentation rates estimated by CRS, C-CRS, and CFCS ^210^Pb dating models.**

| Core profile | Depth (cm) | Mass depth  g cm^-1^ | Calendar Date (CD) yrs. | | | Sedimentation rate | | | |
| --- | --- | --- | --- | --- | --- | --- | --- | --- | --- |
|  |  |  | **CRS** | **C-CRS** | **CFCS** | **CRS** | | **C-CRS** | |
|  |  |  |  |  |  | **MAR gm cm^-2^ yr^-1^** | **SAR**  **cm yr^-1^** | **MAR**  **gm cm^-2^ yr^-1^** | **SAR**  **cm yr^-1^** |
|  |  |  |  |  |  |  |  |  |  |
| C-1 | **4** | **0.16±0.003** | **2016.06±0.40** | **2016.77±0.40** | **2017.51±0.07** | **0.21±0.03** | **1.93±0.30** | **0.33±0.05** | **3.09±0.48** |
|  | **8** | **0.58±0.01** | **2014.21±0.51** | **2015.63±0.51** | **2016.16±0.27** | **0.28±0.03** | **1.87±0.22** | **0.46±0.05** | **3.06±0.35** |
|  | **12** | **1.19±0.02** | **2011.75±0.61** | **2014.15±0.61** | **2014.24±0.55** | **0.22±0.02** | **1.08±0.10** | **0.37±0.03** | **1.82±0.17** |
|  | **16** | **2.00±0.04** | **2006.25±0.84** | **2011.00±0.84** | **2011.66±0.93** | **0.17±0.01** | **0.76±0.07** | **0.31±0.03** | **1.37±0.12** |
|  | **20** | **2.91±0.06** | **2001.24±1.03** | **2006.01±1.04** | **2008.78±1.36** | **0.17±0.02** | **0.95±0.10** | **0.17±0.02** | **0.95±0.10** |
|  | **24** | **3.64±0.07** | **1997.94±1.16** | **2002.72±1.17** | **2006.47±1.70** | **0.19±0.02** | **0.78±0.09** | **0.19±0.02** | **0.78±0.09** |
|  | **28** | **4.60±0.09** | **1990.63±1.45** | **1995.44±1.45** | **2003.43±2.15** | **0.17±0.02** | **0.59±0.07** | **0.17±0.02** | **0.60±0.07** |
|  | **32** | **5.74±0.11** | **1984.69±1.68** | **1989.55±1.68** | **1999.82±2.68** | **0.15±0.02** | **0.62±0.07** | **0.15±0.02** | **0.62±0.07** |
|  | **36** | **6.71±0.13** | **1977.55±1.99** | **1982.46±2.00** | **1996.74±3.13** | **0.14±0.02** | **0.58±0.07** | **0.14±0.02** | **0.59±0.08** |
|  | **40** | **7.68±0.15** | **1971.26±2.28** | **1976.24±2.28** | **1993.68±3.58** | **0.15±0.02** | **0.58±0.09** | **0.15±0.02** | **0.58±0.09** |
|  | **44** | **8.70±0.17** | **1963.49±2.59** | **1968.56±2.60** | **1990.47±4.06** | **0.09±0.01** | **0.38±0.06** | **0.10±0.01** | **0.39±0.06** |
|  | **48** | **9.69±0.19** | **1948.68±3.46** | **1954.00±3.47** | **1987.34±4.52** | **0.06±0.01** | **0.24±0.04** | **0.06±0.01** | **0.25±0.04** |
|  | **52** | **10.65±0.21** | **1932.51±4.67** | **1927.04±4.65** | **1984.30±4.97** | **0.04±0.01** | **0.14±0.03** | **0.03±0.01** | **0.11±0.02** |
| C-2 | **4** | **0.191±0.004** | **2015.53±0.34** | **2015.98±0.34** | **2017.22±0.11** | **0.15±0.01** | **1.28±0.13** | **0.19±0.02** | **1.58±0.16** |
|  | **8** | **0.664±0.01** | **2011.65±0.55** | **2012.87±0.55** | **2015.31±0.37** | **0.13±0.01** | **1.23±0.14** | **0.16±0.02** | **1.55±0.18** |
|  | **12** | **1.088±0.02** | **2009.27±0.69** | **2011.00±0.69** | **2013.58±0.60** | **0.15±0.02** | **1.45±0.20** | **0.20±0.03** | **1.86±0.25** |
|  | **16** | **1.513±0.03** | **2006.72±0.81** | **2008.26±0.81** | **2011.86±0.83** | **0.23±0.02** | **1.03±0.11** | **0.21±0.02** | **0.96±0.10** |
|  | **20** | **2.394±0.05** | **2001.37±1.05** | **2003.23±1.05** | **2008.29±1.32** | **0.20±0.03** | **0.82±0.10** | **0.19±0.02** | **0.77±0.10** |
|  | **24** | **3.365±0.07** | **1997.29±1.25** | **1998.87±1.25** | **2004.35±1.85** | **0.17±0.02** | **0.83±0.11** | **0.16±0.02** | **0.77±0.10** |
|  | **28** | **4.206±0.08** | **1991.52±1.51** | **1992.63±1.51** | **2000.93±2.31** | **0.15±0.02** | **0.74±0.09** | **0.14±0.02** | **0.68±0.08** |
|  | **32** | **5.025±0.10** | **1986.64±1.75** | **1986.00±1.75** | **1997.61±2.76** | **0.14±0.02** | **0.69±0.09** | **0.12±0.01** | **0.60±0.08** |
|  | **36** | **5.813±0.12** | **1979.64±2.12** | **1978.64±2.12** | **1994.42±3.20** | **0.11±0.01** | **0.44±0.06** | **0.10±0.01** | **0.42±0.05** |
|  | **40** | **6.773±0.14** | **1967.10±2.82** | **1965.20±2.82** | **1990.52±3.72** | **0.09±0.01** | **0.33±0.05** | **0.08±0.01** | **0.30±0.04** |
|  | **44** | **7.871±0.16** | **1956.95±3.63** | **1954.00±3.63** | **1986.07±4.33** | **0.10±0.02** | **0.29±0.05** | **0.09±0.02** | **0.26±0.05** |
|  | **Continue Table 2S:** | | | | | | | | |
|  | **48** | **9.255±0.19** | **1936.58±4.58** | **1946.48±4.58** | **1980.45±5.09** | **0.07±0.02** | **0.22±0.05** | **0.09±0.02** | **0.29±0.07** |
|  | **52** | **10.436±0.21** | **1927.42±4.84** | **1937.03±4.84** | **1975.66±5.74** | **0.04±0.01** | **0.18±0.04** | **0.06±0.01** | **0.25±0.06** |
| C-3 | **4** | **0.24±0.01** | **2016.61±0.25** | **2016.72±0.25** | **2017.00±0.17** | **0.33±0.04** | **2.08±0.27** | **0.35±0.05** | **2.26±0.29** |
|  | **8** | **0.87±0.02** | **2014.05±0.49** | **2014.37±0.49** | **2014.38±0.62** | **0.23±0.03** | **1.28±0.16** | **0.25±0.03** | **1.40±0.17** |
|  | **12** | **1.59±0.03** | **2010.34±0.73** | **2011.00±0.73** | **2011.36±1.15** | **0.21±0.02** | **1.06±0.13** | **0.23±0.03** | **1.17±0.14** |
|  | **16** | **2.38±0.05** | **2006.80±0.90** | **2006.44±0.90** | **2008.03±1.72** | **0.23±0.02** | **0.94±0.10** | **0.16±0.02** | **0.65±0.07** |
|  | **20** | **3.38±0.07** | **2001.55±1.11** | **1998.23±1.11** | **2003.86±2.44** | **0.15±0.01** | **0.65±0.07** | **0.10±0.01** | **0.42±0.04** |
|  | **24** | **4.30±0.09** | **1994.64±1.41** | **1986.00±1.40** | **1999.99±3.11** | **0.13±0.01** | **0.45±0.05** | **0.07±0.01** | **0.24±0.03** |
|  | **28** | **5.46±0.11** | **1984.76±1.83** | **1982.81±1.83** | **1995.15±3.94** | **0.13±0.01** | **0.34±0.04** | **0.09±0.01** | **0.22±0.02** |
|  | **32** | **6.98±0.14** | **1969.87±2.69** | **1960.12±2.67** | **1988.80±5.04** | **0.10±0.01** | **0.25±0.03** | **0.05±0.01** | **0.13±0.02** |
|  | **36** | **8.63±0.17** | **1954.51±3.68** | **1951.06±3.67** | **1981.89±6.23** | **0.12±0.02** | **0.28±0.05** | **0.08±0.01** | **0.18±0.03** |
|  | **40** | **10.34±0.21** | **1943.29±4.28** | **1947.47±4.29** | **1974.72±7.47** | **0.11±0.02** | **0.27±0.06** | **0.09±0.02** | **0.21±0.04** |
|  | **44** | **11.99±0.24** | **1919.71±6.94** | **1943.51±7.02** | **1967.82±8.66** | **0.05±0.01** | **0.12±0.03** | **0.07±0.02** | **0.18±0.05** |
| C-4 | **8** | **0.41±0.01** | **2015.11±0.43** | **2014.26±0.43** | **2016.35±0.43** | **0.17±0.02** | **1.45±0.19** | **0.13±0.02** | **1.11±0.14** |
|  | **12** | **0.87±0.02** | **2012.64±0.62** | **2011.00±0.62** | **2014.46±0.92** | **0.16±0.02** | **1.41±0.19** | **0.12±0.02** | **1.05±0.14** |
|  | **16** | **1.34±0.03** | **2009.53±0.82** | **2007.68±0.82** | **2012.57±1.42** | **0.18±0.02** | **1.28±0.16** | **0.17±0.02** | **1.20±0.15** |
|  | **20** | **1.90±0.04** | **2006.41±0.97** | **2004.32±0.97** | **2010.27±2.02** | **0.19±0.02** | **1.18±0.15** | **0.18±0.02** | **1.09±0.14** |
|  | **24** | **2.56±0.05** | **2002.82±1.18** | **2000.44±1.18** | **2007.61±2.71** | **0.18±0.02** | **0.96±0.13** | **0.17±0.02** | **0.88±0.12** |
|  | **28** | **3.32±0.07** | **1997.86±1.46** | **1995.00±1.46** | **2004.49±3.53** | **0.12±0.01** | **0.63±0.08** | **0.11±0.01** | **0.57±0.07** |
|  | **32** | **4.07±0.08** | **1989.83±1.95** | **1986.00±1.95** | **2001.46±4.32** | **0.08±0.01** | **0.43±0.06** | **0.11±0.01** | **0.58±0.08** |
|  | **36** | **4.86±0.10** | **1978.68±2.72** | **1963.27±2.70** | **1998.26±5.15** | **0.08±0.01** | **0.33±0.05** | **0.08±0.01** | **0.32±0.05** |
|  | **40** | **5.82±0.12** | **1966.52±3.83** | **1952.81±3.81** | **1994.36±6.17** | **0.08±0.02** | **0.38±0.07** | **0.08±0.02** | **0.38±0.07** |
|  | **44** | **6.64±0.13** | **1958.69±4.69** | **1948.87±4.67** | **1991.02±7.04** | **0.07±0.02** | **0.26±0.06** | **0.08±0.02** | **0.30±0.07** |
|  | **48** | **7.74±0.15** | **1925.90±6.29** | **1941.26±6.34** | **1986.53±8.22** | **0.02±0.01** | **0.08±0.02** | **0.05±0.01** | **0.20±0.05** |

**
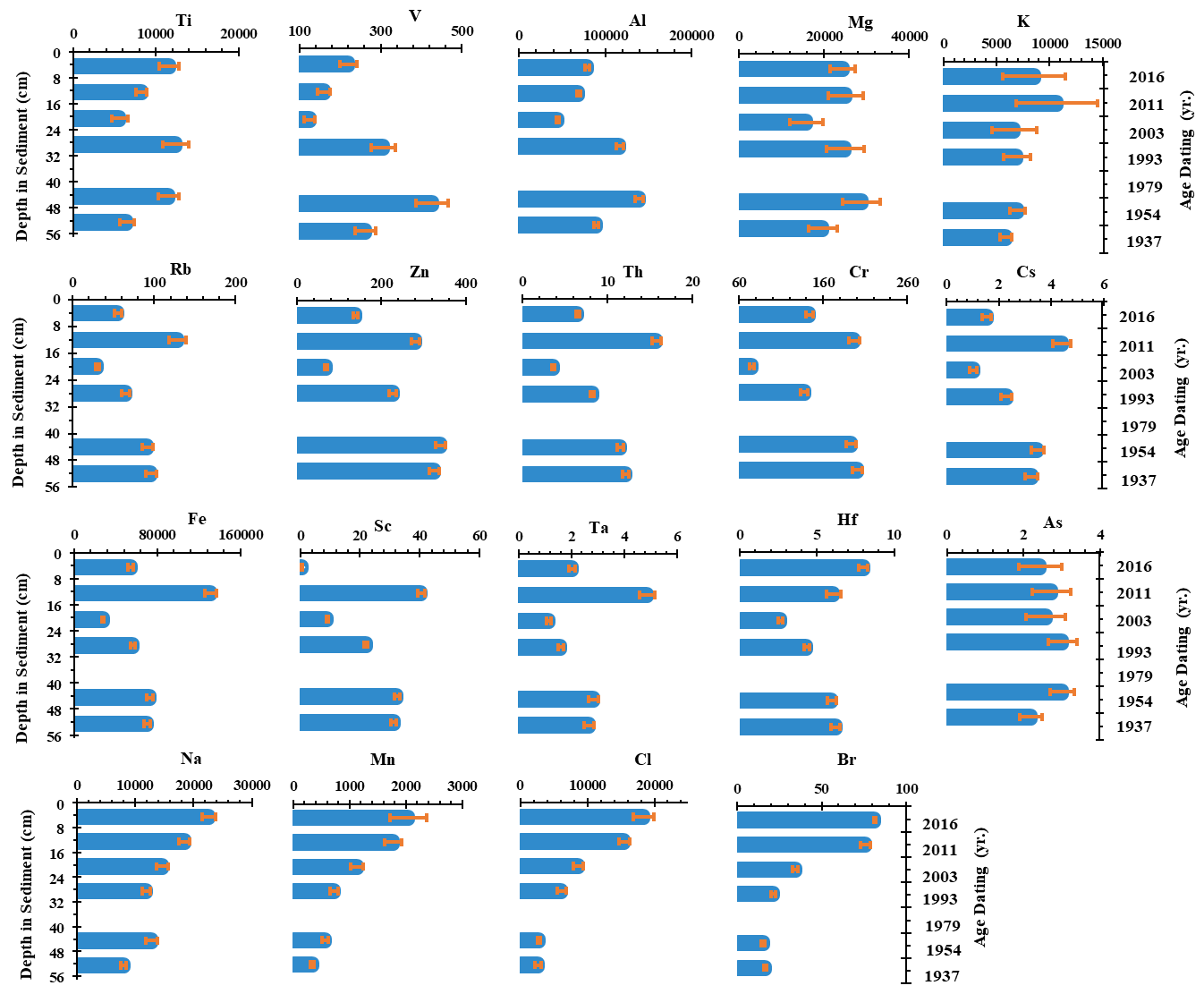
**

**Fig. 1S: Vertical distribution of metals concentration in the core samples C-2.**

**
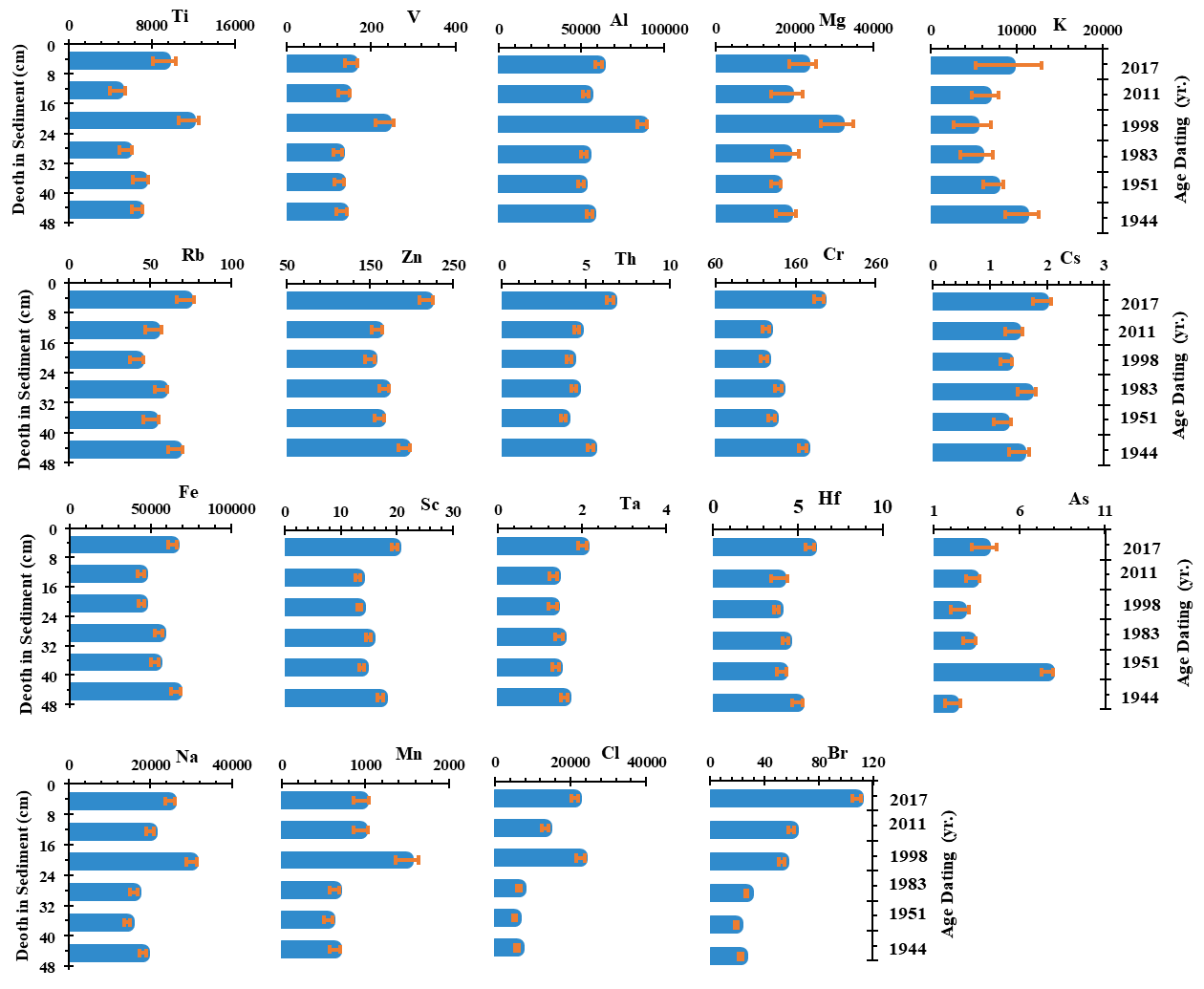
**

**Fig.2S: Vertical distributions of metals concentrations in the core samples C-3.**


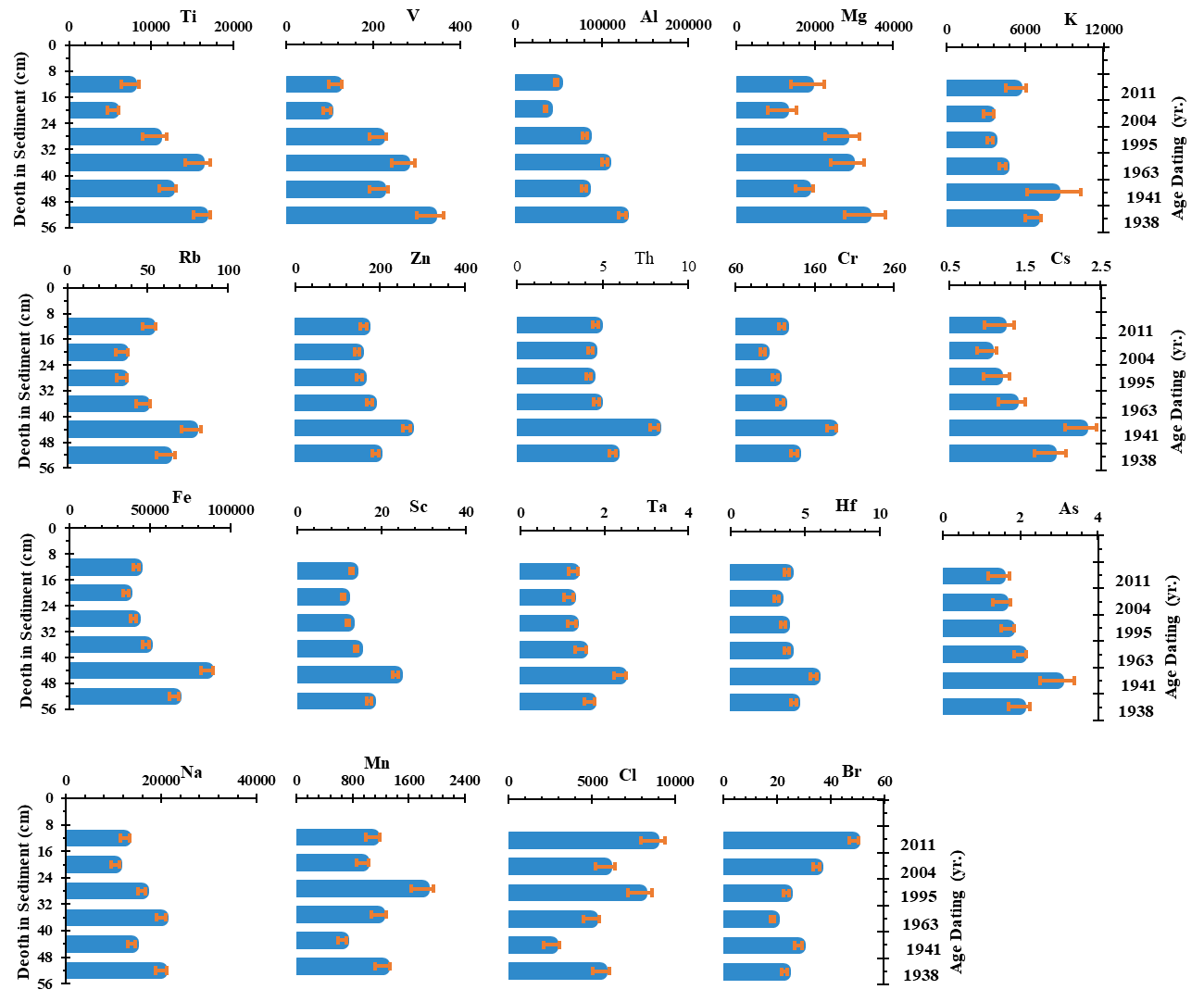


**Fig. 3S: Vertical distribution of metals concentration in the core samples C-4**
